# Genome-scale CRISPR/Cas9 screen determines factors modulating sensitivity to ProTide NUC-1031

**DOI:** 10.1038/s41598-019-44089-3

**Published:** 2019-05-21

**Authors:** Awa Sarr, Jennifer Bré, In Hwa Um, Tsz Huen Chan, Peter Mullen, David J. Harrison, Paul A. Reynolds

**Affiliations:** 1 School of Medicine, University of St Andrews, St Andrews, KY16 9TF UK; 2 Biomedical Sciences Research Complex, University of St. Andrews, St. Andrews, UK

**Keywords:** Predictive markers, Pancreatic cancer

## Abstract

Gemcitabine is a fluoropyrimidine analogue that is used as a mainstay of chemotherapy treatment for pancreatic and ovarian cancers, amongst others. Despite its widespread use, gemcitabine achieves responses in less than 10% of patients with metastatic pancreatic cancer and has a very limited impact on overall survival due to intrinsic and acquired resistance. NUC-1031 (Acelarin), a phosphoramidate transformation of gemcitabine, was the first anti-cancer ProTide to enter the clinic. We find it displays important *in vitro* cytotoxicity differences to gemcitabine, and a genome-wide CRISPR/Cas9 genetic screening approach identified only the pyrimidine metabolism pathway as modifying cancer cell sensitivity to NUC-1031. Low deoxycytidine kinase expression in tumour biopsies from patients treated with gemcitabine, assessed by immunostaining and image analysis, correlates with a poor prognosis, but there is no such correlation in tumour biopsies from a Phase I cohort treated with NUC-1031.

## Introduction

NUC-1031 (Acelarin), a phosphoramidate transformation of gemcitabine, is the first anti-cancer ProTide to enter the clinic^1^. Analogues of cytidine are the backbone of many therapeutic regimens in oncology. Historically, Ara-C (Cytarabine) and more recently, gemcitabine^2^, are first-line chemotherapy agents used in patients with pancreatic cancer^3^ and in combination treatments for ovarian, breast, biliary tract, lung, and bladder cancers^4,5^. Gemcitabine acts as a cytotoxic agent primarily by blocking DNA synthesis in cancer cells^6–8^. It is imported into cells through membrane transporters, including human Equilibrative Nucleotide Transporter 1 (hENT1), decreased expression of which in pancreatic cancer may be associated with poor overall survival^9^. Once inside the cell, gemcitabine requires phosphorylation to difluorodeoxycytidine monophosphate (dFdCMP) by deoxycytidine kinase (DCK), which represents the rate-limiting step for further phosphorylation to the active diphosphate (dFdCDP) and triphosphate (dFdCTP) metabolites^2^. Of these, dFdCTP is the more active and incorporates into DNA to inhibit its synthesis. Subsequent failure of DNA repair triggers apoptosis and inhibits tumour growth^10,11^. dFdCDP inactivates ribonucleotide reductase, depleting the deoxyribonucleotide pools necessary for DNA synthesis, potentiating the effects of dFdCTP^12,13^. Gemcitabine is also rapidly catabolized by cytidine deaminase (CDA) generating difluorodeoxyuridine (dFdU)^14^.

Despite its widespread use, gemcitabine achieves responses in less than 10% of patients with metastatic pancreatic cancer and has very limited impact on overall survival^15^. Many cancers have an innate resistance to gemcitabine or, once exposed to gemcitabine, develop resistance, often within weeks of treatment initiation, markedly limiting its efficacy and clinical benefit^8,16^. Three key cancer cell resistance mechanisms have been associated with a poor survival prognosis for gemcitabine: transport, activation, and breakdown. Cells deficient in the nucleoside transporter hENT1 are highly resistant to gemcitabine^17^ and patients with pancreatic cancer who express low or undetectable levels of hENT1 have significantly lower median survival times than those with detectable levels^18^. Deficiency of the activating enzyme DCK led to acquired gemcitabine resistance in a human ovarian carcinoma cell line exposed to increasing levels of gemcitabine *in vitro*^19^ and patients with pancreatic cancer who express low levels of DCK have significantly poorer overall survival than those with high levels^20^. Finally, increased levels of the catabolising enzyme CDA have been associated with reduced median survival times in gemcitabine-treated patients with pancreatic cancer^21^.

The ProTide drug NUC-1031 is comprised of a pre-activated nucleotide analogue (gemcitabine monophosphate) and a protective phosphoramidate moiety, which is a specific combination of aryl, ester, and amino acid groupings. Pre-clinical data show the increased lipophilicity of NUC-1031 enables it to circumvent hENT1-mediated transmembrane transport and, once inside the cell, the phosphoramidate protective group is cleaved off by esterases, releasing dFdCMP which is then rapidly converted to dFdCDP and dFdCTP, bypassing the rate-limiting step of phosphorylation by DCK. Furthermore, NUC-1031 avoids CDA-mediated catabolism, thus preventing dFdU accumulation^22–24^. Both nucleotide synthesis and degradation are important in maintaining the dNTP pool and substrate cycling by 5ʹ‐nucleotidases and nucleoside kinases represent points of control^25^.

Genome-scale genetic knockdown screens have been successfully used to identify genes involved in drug resistance/sensitivity for a variety of chemotherapeutic agents^26^ and we employed this approach to screen for candidate genes mediating cancer cell sensitivity to NUC-1031. Surprisingly, the only pathway consistently selected was pyrimidine metabolism; specifically, multiple sgRNAs targeting two genes, DCK and deoxycytidine triphosphate pyrophosphate 1 (DCTPP1), involved in the maintenance of the dCMP/dCTP pool. In contrast, there were no consistent hits selected from the gemcitabine screen. We show that, although similar in structure, NUC-1031 displays important *in vitro* cytotoxicity differences to gemcitabine. While we find low DCK expression in tumour biopsies from patients treated with gemcitabine, assessed by immunostaining and image analysis, correlates with a poor prognosis, we find no such correlation in tumour biopsies from a Phase I cohort treated with NUC-1031. These data suggest that in contrast to gemcitabine, low DCK expression should not preclude patients from consideration for NUC-1031 treatment and that DCK is not a predictive marker of clinical response to NUC-1031.

## Results

### Exogenous dCyd confers complete resistance to gemcitabine while sensitivity to NUC-1031 is retained

**Figure 1:**
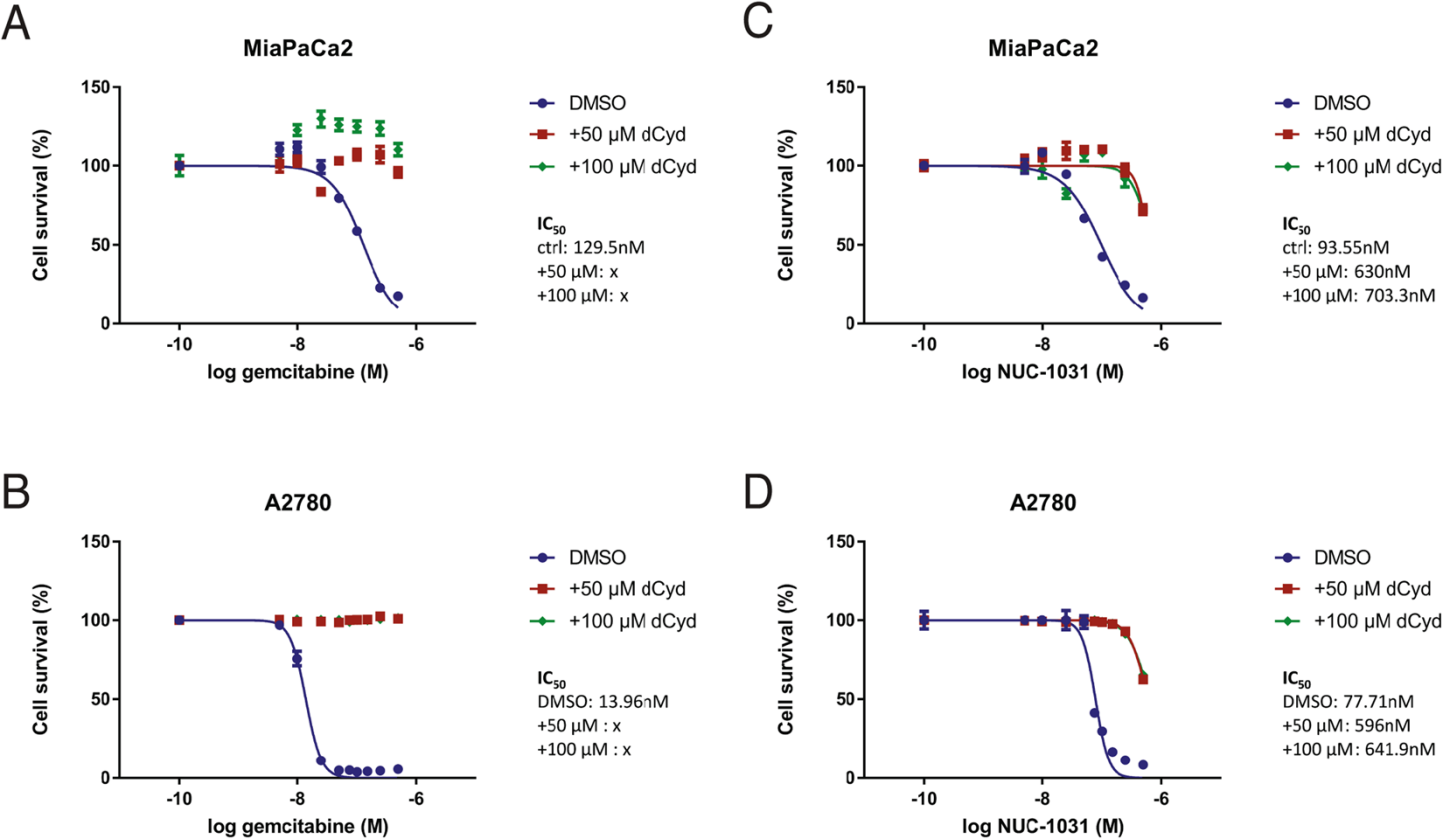
Exogenous dCyd negates efficacy of gemcitabine but not NUC-1031. (**A**,**B**) Dose-response curves for MiaPaCa2 or A2780 cells 4d after treatment with gemcitabine or (**C**,**D**) NUC-1031 and simultaneous addition of either DMSO, 50 µM or 100 µM of deoxycytidine (dCyd). Values represent mean +/− SEM (n = 6). A2780 vs A2780 + 50 µM dCyd vs A2780 + 100 µM dCyd: p = 0.0022; Mann-Whitney test).

In order to investigate the effect of NUC-1031 and gemcitabine on dCMP/dCTP pool regulation, cytotoxicity assays for NUC-1031 and gemcitabine were carried out on MiaPaCa2 pancreatic cancer cells and A2780 ovarian cancer cells. In the presence of deoxycytidine (dCyd) to competitively inhibit DCK, MiaPaCa2 and A2780 cells (Fig. 1A,B) were completely resistant to gemcitabine, confirming the requirement of DCK for gemcitabine activation. By contrast, at equal concentrations of dCyd, NUC-1031 retained its cytotoxicity (Fig. 1C,D), albeit showing a modest decrease (30–35% reduction at equimolar doses). While dCyd does partially impair NUC-1031 activity, the effect was much less than the complete inhibition seen with gemcitabine. Pre-treatment of cells with dCyd before the addition of NUC-1031 also showed similar results (Fig. S1). These data are consistent with the phosphorylated status of NUC-1031, compared to gemcitabine.

To further elucidate these differences, cell cycle analysis was performed on A2780 cells treated at the IC_50_ dose of NUC-1031 or gemcitabine for 2 h, followed by media washout. At 24 h after washout, more A2780 cells were in S phase after treatment with gemcitabine (62.1%) or NUC-1031 (57.85%) compared to DMSO-treated control (36.85%) (Fig. S2). However, at 48 h after washout, more A2780 cells were arrested in S phase after treatment with NUC-1031 (50.65%) than after treatment with gemcitabine (40.05%) or DMSO (36.55%) (Fig. S2). At 72 h after washout, more A2780 cells were in G2/M phase after treatment with NUC-1031 (18.95%) than after treatment with gemcitabine (12.6%) or DMSO (8.13%) (Fig. S2). Taken together, these data suggest that NUC-1031 and gemcitabine display important *in vitro* cytotoxicity differences and that the effects of NUC-1031 persist for longer *in vitro*, compared to gemcitabine.

### Genome-Scale CRISPR/Cas9 screen implicates pyrimidine metabolism in NUC-1031 sensitivity

**Figure 2:**
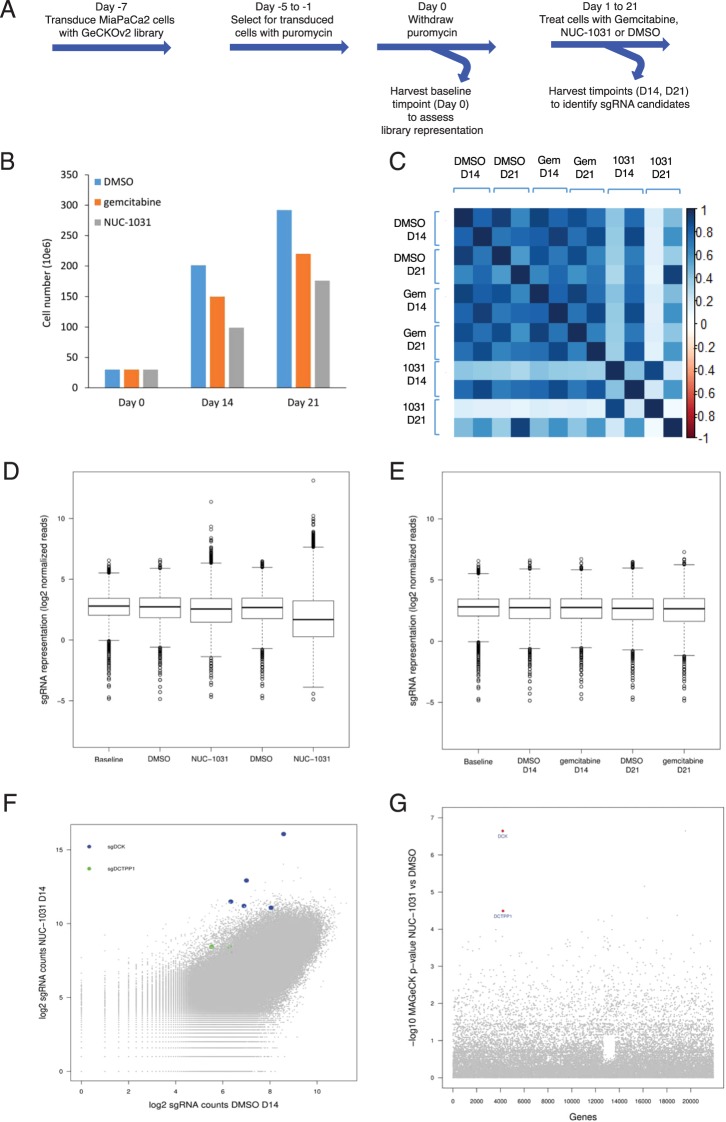
Genome-Scale CRISPR/Cas9 Screen Implicates DCK and DCTPP1 in NUC-1031 sensitivity. (**A**) Experimental design of the GECKOv2 screen for gemcitabine and NUC-1031 resistance, performed in MiaPaCa2 cells, in 2 biological replicates. (**B**) MiaPaCa2 cell number at d0, d14 and d21 after treatment with either dimethyl sulfoxide (DMSO) as control, gemcitabine or NUC-1031. (**C**) Heat map of sgRNA abundance comparing biological replicates and treatment conditions. (**D**,**E**) Distribution of sgRNA reads frequency before treatment (Baseline), in DMSO (Control), NUC-1031 or gemcitabine treated cells after d14 and d21 of exposure. The box extends from the first to the third quartile with the whiskers denoting 1.5 times the interquartile range and show an increased variability of sgRNA frequency in cells treated with NUC-1031 compared DMSO treated cells. (**F**) Scatterplot of sgRNA read counts in NUC-1031 treated cells compared to control (DMSO) cells showing enrichment of DCK and DCTPP1 sgRNAs after 14d exposure. (**G**) Identification of candidate genes, targeted by enriched sgRNAs, in NUC-1031 treated cells compared to control cells, using MAGeCK p-value analysis.

To identify genes involved in modulating resistance/sensitivity to NUC-1031 or gemcitabine, the GeCKOv2 genome-scale CRISPR/Cas9 knockdown library^27,28^ was used in pancreatic MiaPaCa2 cells and sgRNA distribution compared by next generation sequencing after 14d and 21d of drug treatment (Fig. 2A; Fig. S3). Exposure to either NUC-1031 or gemcitabine resulted in retarded population growth of transduced MiaPaCa2 cells (Fig. 2B), therefore enabling the enrichment of a small group of cells that were rendered more drug-resistant by Cas9:sgRNA-mediated modification. After 14d and 21d of NUC-1031 treatment, the sgRNA distribution was significantly different when compared to DMSO (vehicle)-treated cells, particularly after 21d, as well as an increased variability, illustrated by a larger interquartile range, indicating the selection of specific sgRNAs in response to the treatment (Fig. 2C; p < 2.2 × 10^−16^, Mann-Whitney/Wilcoxon rank sum test). Interestingly, gemcitabine treatment induced a smaller but statistically significantly different sgRNA distribution, compared to DMSO-treated cells (Fig. 2C; p = 0.001117 at d14, p < 2.2 × 10^−16^ at d21; Mann-Whitney/Wilcoxon rank sum test). For a subset of genes, there was enrichment of multiple sgRNAs that target each gene after 14d and 21d of NUC-1031 treatment (Fig. 2D), suggesting that loss of these particular genes contributes to increased NUC-1031 resistance. In contrast, there were no consistent hits from the gemcitabine screen (Fig. 2E, Table S1). The MAGeCK algorithm^29^ was used to rank screening hits by the consistent enrichment among multiple sgRNAs targeting the same gene (Fig. 2F,G). The highest-ranking genes included the previously reported gemcitabine resistance factor DCK^30^ and also several other genes, including DCTPP1, implicated in modulating intracellular dCTP^31^ (Table 1). These hits were also identified through a second independent library transduction (Fig. S4, Table S1).

**Table 1:** Top ranked genes from MAGeCK analysis.

MAGeCK rank	gemcitabine				NUC-1031			
	Negative selection		Positive selection		Negative selection		Positive selection	
	Day 14	Day 21	Day 14	Day 21	Day 14	Day 21	Day 14	Day 21
1	CSF3R	NAA10	PP1R32	TSC2	TSC2	hsa-mir-555	TMEM165	TMEM165
2	LRRC24	POLR2D	hsa-mir-4711	CRYGC	ZBTB46	TSC2	DCK	DCK
3	POLB	PELO	NCOA3	ATRNL1	TSC1	DMPK	PYCR2	NTC_0397
4	MBLAC2	RPL14	CENPE	SH3BP4	ZNF281	PCOLCE	DCTPP1	SIRPD
5	NPBWR1	HPS5	UGGT2	hsa-mir-6727	COL4A1	OR10A5	SYNCRIP	TSPAN12
6	COG7	HM13	ARL1	MAP4K5	DDIT4	RUFY1	NTC_0731	HCN3
7	CTTNBP2	WDR43	hsa-mir-548h-5	hsa-mir-1254-2	OR51E2	hsa-mir-185	RASSF7	POMP
8	TNFRSF1A	COX5A	UBXN2A	NTC_0555	PHTF1	OR1M1	NACC2	NTC_0203
9	WWP2	XRCC5	NADK2	hsa-mir-1265	ERBB2IP	CCDC42B	NTC_0040	NTC_0045
10	ATP6V1B1	KIAA1239	CLEC19A	CDH29	LHPP	KCNA3	COQ4	DDO

Genes ranked by MAGeCK by either negative selection or positive selection in gemcitabine or NUC-1031 treated cells after d14 and d21 of exposure.

### Validation of candidate genes

**Figure 3:**
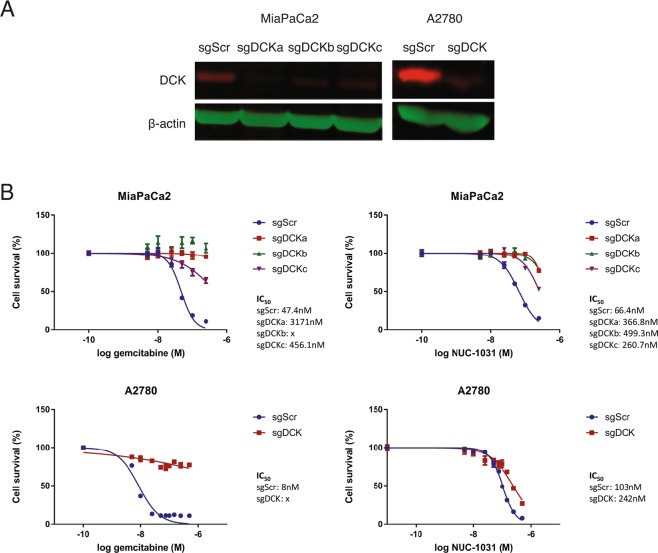
DCK mediates NUC-1031 sensitivity in pancreatic and ovarian cancer cells. (**A**) DCK protein expression analyzed by Western blot in MiaPaCa2 and A2780 cells transduced with independent sgRNAs targeting DCK (sgDCK) or a non-targeting scrambled control sgRNA (sgScr). (**B**) Dose-response curves for MiaPaCa2 and A2780 cells transduced with individual sgRNAs targeting DCK or a non-targeting scrambled control sgRNA (sgScr) and treated with NUC-1031 or gemcitabine (n = 6, +/−SEM. MiaPaCa2 sgScr vs MiaPaCa2 sgDCK: p = 0.0022, A2780 sgScr vs A2780 sgDCK: p = 0.0022 for NUC-1031 and p = 0.0043 for gemcitabine; Mann-Whitney test).

Top-ranking genes from the GeCKOv2 screen were validated individually using independent sgRNAs cloned into pLentiCRISPRv2 and transduced into MiaPaCa2 cells in order to generate distinct knockdown cell lines for each gene. For DCK, knockdown efficiency was assessed by Western blot and DCK expression was found reduced by more than 70% in the MiaPaCa2 knockdown cell lines compared to MiaPaCa2-sgScr cells (Fig. 3A). Interestingly, the sgRNAs conferred a 4.5 to 7-fold increased resistance to NUC-1031 in MiaPaCa2-sgDCK cells, compared to MiaPaCa2-sgScr cells (Fig. 3B). However, at NUC-1031 concentrations of 250 nM, there was approximately 34% death (range of 21–47% for the 3 sgRNAs), compared to MiaPaCa2-sgDCK cells treated with gemcitabine that survived and were completely resistant to 250 nM gemcitabine (Fig. 3B). Likewise, ovarian A2780-sgDCK cells treated with gemcitabine were completely resistant to 500 nM gemcitabine, whereas A2780-sgDCK cells treated with NUC-1031 were 2-fold more resistant (IC_50_ sgDCK 242 nM vs IC_50_ sgScr 103 nM; p = 0.0022), compared to A2780-sgScr cells (Fig. 3B).

**Figure 4:**
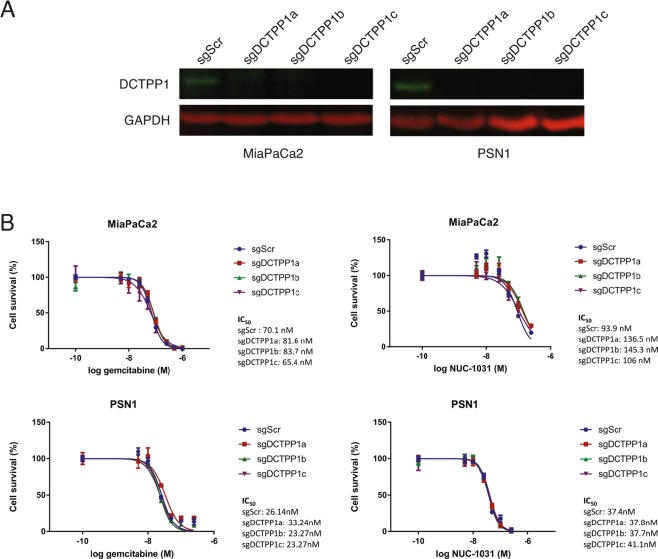
DCTPP1 mediates NUC-1031 sensitivity in pancreatic cancer cells. (**A**) DCTPP1 protein expression analyzed by Western blot in MiaPaCa2 and PSN1 cells transduced with independent sgRNAs targeting DCTPP1 (sgDCTPP1) or a non-targeting scrambled control sgRNA (sgScr). (**B**) Dose-response curves for MiaPaCa2 and PSN1 cells transduced with individual sgRNAs targeting DCTPP1 or a non-targeting scrambled control sgRNA (sgScr) and treated NUC-1031 or gemcitabine (n = 6, +/−SEM. For NUC-1031: MiaPaCa2 sgScr vs MiaPaCa2 sgDCTPP1a: p = 0.0022; MiaPaCa2 sgScr vs MiaPaCa2 sgDCTPP1b: p = 0.0087; MiaPaCa2 sgScr vs MiaPaCa2 sgDCTPP1c: p = 0.0152;, for gemcitabine: MiaPaCa2 sgScr vs MiaPaCa2 sgDCTPP1a: p = 0.0649; MiaPaCa2 sgScr vs MiaPaCa2 sgDCTPP1b: p = 0.3939; MiaPaCa2 sgScr vs MiaPaCa2 sgDCTPP1c: p = 0.8182 and PSN1 sgScr vs PSN1 sgDCTPP1a: p = 0.1797; PSN1 sgScr vs PSN1 sgDCTPP1b: p = 0.8182; PSN1 sgScr vs PSN1 sgDCTPP1c: p = 0.3939; Mann-Whitney test).

For DCTPP1, knockdown efficiency was assessed by Western blot analysis and DCTPP1 expression was found to be reduced by more than 90% in the MiaPaCa2 knockdown cell lines compared to MiaPaCa2-sgScr cells (Fig. 4A). Interestingly, the DCTPP1 sgRNAs conferred a 1.5-fold decreased sensitivity to NUC-1031 in MiaPaCa2-sgDCTPP1 cells, compared to MiaPaCa2-sgScr cells, indicating a small but consistent decrease in MiaPaCa2 cells sensitivity to NUC-1031 in the absence of DCTPP1 (Fig. 4B). On the contrary, no change in sensitivity was detected in MiaPaCa2-sgDCTPP1 cells compared to MiaPaCa2-sgScr cells in response to gemcitabine treatment (Fig. 4B). No change in sensitivity was detected for NUC-1031 or gemcitabine in pancreatic PSN1-sgDCTPP1 cells, compared to PSN1-sgScr cells (Fig. 4B). These data suggest that the modulatory effect of DCTPP1 on NUC-1031 sensitivity is specific to MiaPaCa2 cells.

### Simultaneous knockdown of DCTPP1 and DCK shows no synergy

**Figure 5:**
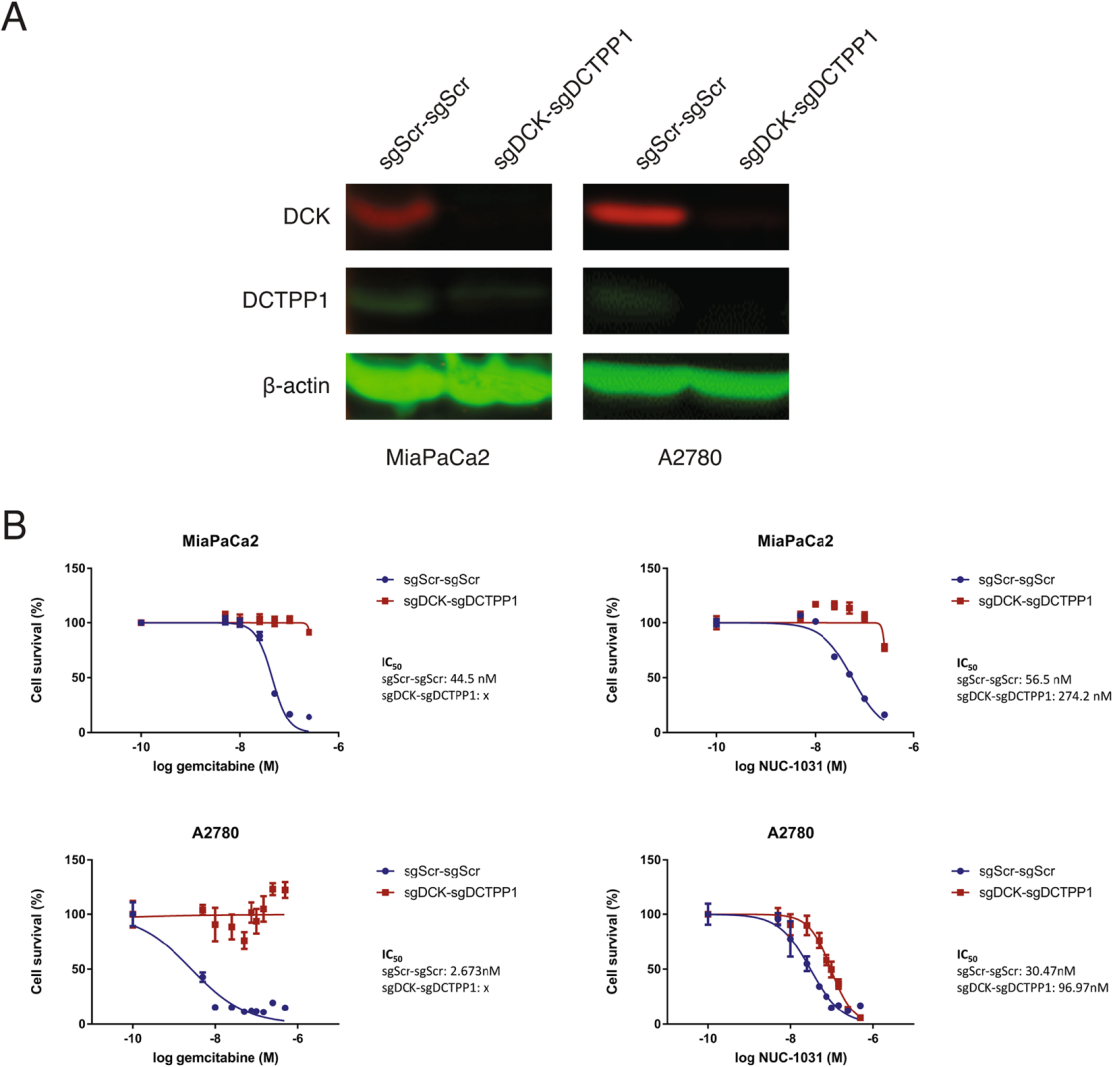
DCK and DCTPP1 simultaneous knockdown induced no synergistic effect in pancreatic and ovarian cancer cells to NUC-1031. (**A**) DCK and DCTPP1 protein expression analyzed by Western blot in MiaPaCa2 and A2780 cells transfected with a pX333 plasmid containing sgRNA sequences targeting DCK (sgDCK) and DCTPP1 (sgDCTPP1) or a non-targeting scrambled control sgRNA (sgScr). (**B**) Dose response curves for MiaPaCa2 and A2780 sgDCK-sgDCTPP1 cells and sgScr-sgScr cells (control), 4d after treatment with NUC-1031 or gemcitabine (n = 6, +/−SEM. MiaPaCa2 sgScr-sgScr vs MiaPaCa2 sgDCKe-sgDCTPP1a: p = 0.0022, A2780 sgScr vs A2780 sgDCK-sgDCTPP1: p = 0.0022; Mann-Whitney test).

Since both DCK and DCTPP1 are involved in pyrimidine metabolism and maintenance of the dCMP pool, simultaneous inactivation of DCK and DCTPP1 was performed to assess for any synergistic effects, compared with individual knockdown. One guide RNA targeting DCK (sgDCK) and one targeting DCTPP1 (sgDCTPP1) or two non-targeting guide RNAs (sgScr), were cloned into pX333. Control (pX333 sgScr-sgScr) and double knockdown (pX333 sgDCK-sgDCTPP1) plasmids were introduced independently into MiaPaCa2 and A2780 cells by nucleofection. DCK protein expression was reduced by approximately 86% and DCTPP1 by approximately 72% in MiaPaCa2-sgDCK-sgDCTPP1 cells (Fig. 5A). MiaPaCa2-sgDCK-sgDCTPP1 cells displayed a 5-fold decreased sensitivity to NUC-1031, compared to MiaPaCa2-sgScr-sgScr cells, while MiaPaCa2-sgDCK-sgDCTPP1 cells were completely resistant to gemcitabine (Fig. 5B). Similar results were observed in A2780 cells with decrease of protein expression by 85% and 90% for DCK and DCTPP1, respectively. A2780-sgDCK-sgDCTPP1 cells displayed a 3-fold decreased sensitivity to NUC-1031, compared to A2780-sgScr-sgScr cells, while A2780-sgDCK-sgDCTPP1 cells were completely resistant to gemcitabine (Fig. 5B). These data suggest there is no synergistic effect on NUC-1031 sensitivity by simultaneously reducing both DCK and DCTPP1 expression.

### DCK expression is not predictive in patients treated with NUC-1031

**Figure 6:**
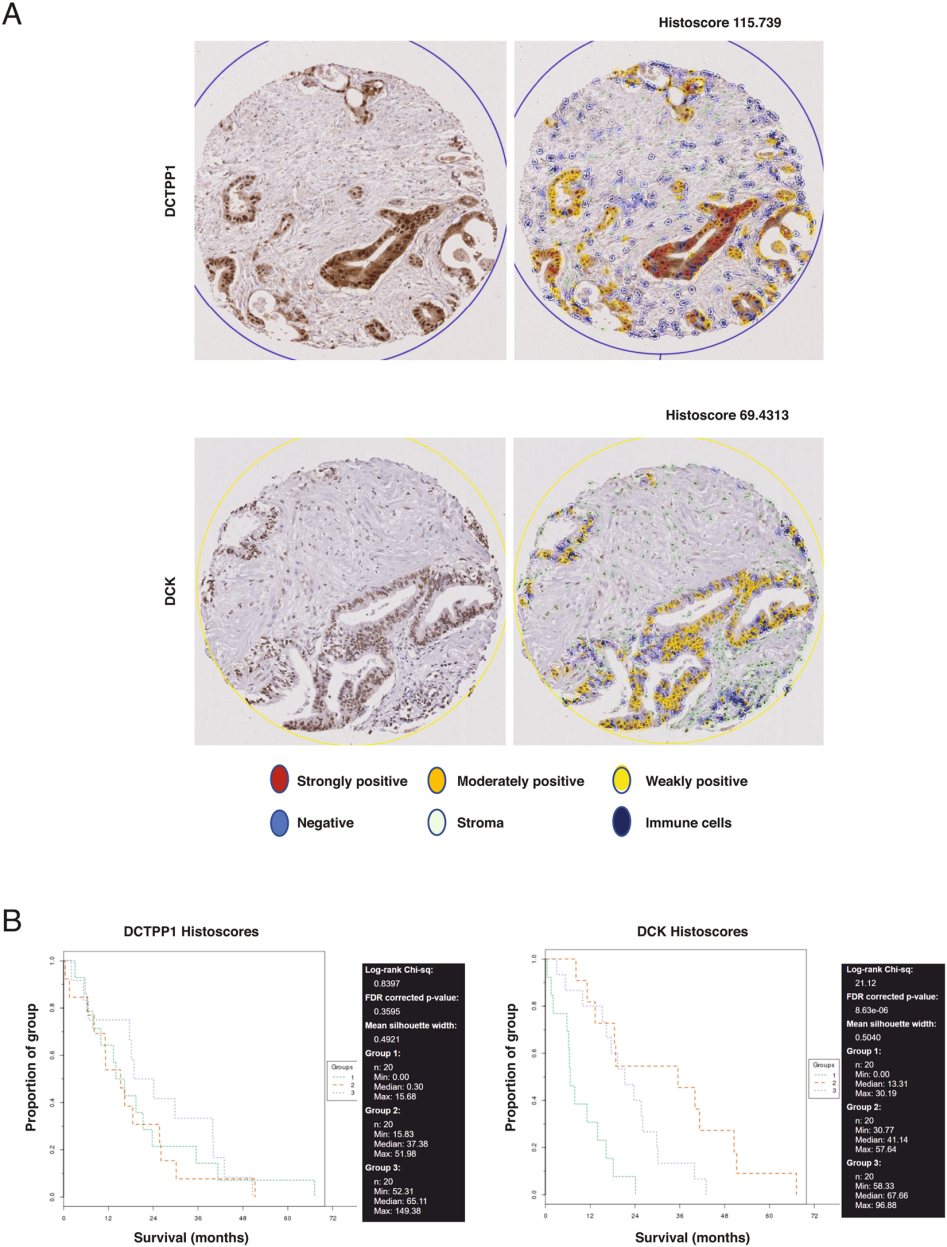
DCK expression is associated with outcome in gemcitabine-treated patients. (**A**) DCTPP1 expression in one tissue core before (left panel) and after (right panel) QuPath analysis based on staining intensity in tumour cells. A histoscore of 115.739 was calculated based on the proportion of positive cells and their staining intensity. DCK expression in a second tissue core before (left panel) and after (right panel) QuPath analysis based on staining intensity in tumour cells. A histoscore of 69.4313 was calculated based on the proportion of positive cells and their staining intensity. (**B**) KM survival curves for DCTPP1 and DCK expression generated using TMA Navigator. All of the tissue cores were divided in three groups according to their histoscore. Each group was composed of 20 samples presenting histoscores in the indicated ranges. Survival of patients is presented in months.

In order to assess the clinical relevance of DCK and DCTPP1 expression, cancer biopsy tissues from either pancreatic cancer patients who received gemcitabine/did not receive chemotherapy or from patients from a pan-cancer Phase I cohort treated with NUC-1031 were immunostained using antibodies to DCK and DCTPP1, and scanned images obtained from Zeiss AxioScan were quantified using QuPath^32^. After review by a pathologist (DJH), 60 pancreatic cancer tissue microarray (TMA) cores were identified as containing tumours, and these had associated survival data. DCTPP1 was expressed in 50 out of 60 pancreatic cancer cores, mainly in tumour cells that displayed mostly nuclear localization but also in cells in the tumour microenvironment, where DCTPP1 was localized in both the cytoplasm and nucleus. DCK was expressed in 55 out of 60 pancreatic cancer cores, in the cytoplasm and strongly in the nucleus, not only in tumour cells, but also in stromal and immune cells in the tumour microenvironment (Fig. 6A). To determine whether DCTPP1 and DCK expression levels were associated with patient outcome, Kaplan-Meier (KM) analyses were performed on histoscores using TMA navigator (www.tmanavigator.org^33^). KM analysis showed that there was no significant difference in survival (FDR corrected p-value = 0.3595) between those patients with low DCTPP1 expression (histoscores 0 to 15.68), those with medium DCTPP1 expression (histoscores 15.83 to 51.98) and those with high DCTPP1 expression (histoscores 52.31 to 149.38) (Fig. 6B). However, as expected from previous studies, KM analysis showed that patients with low DCK expression (histoscores 0 to 30.19), had a significantly shorter survival (FDR corrected p-value = 8.63 × 10^−6^) than those with medium DCK expression (histoscores 30.77 to 57.64) or high DCK expression (histoscores 58.33 to 96.88) (Fig. 6B).

**Figure 7:**
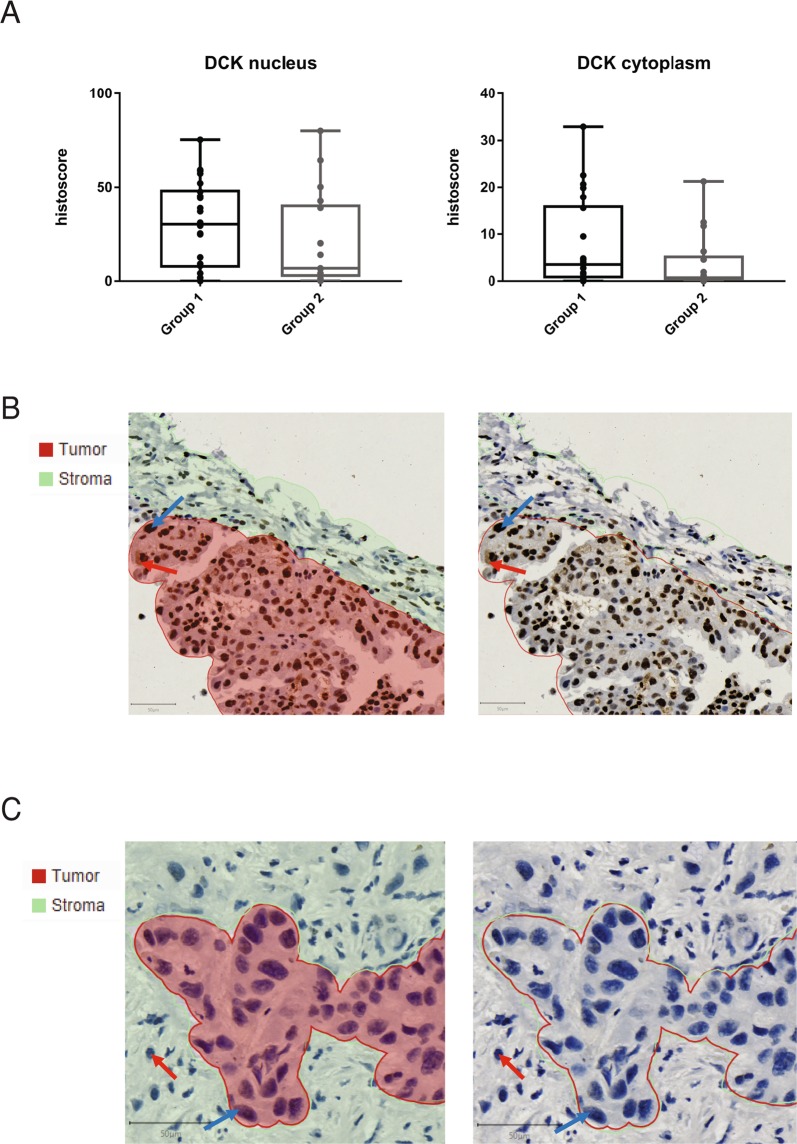
DCK expression in archival tissue does not correlate with outcome in patients treated with NUC-1031. (**A**) Nucleus and cytoplasm DCK histoscores in tissue from patients who achieved stable disease for less than six months, or progressive disease developed within that time (group 1, n = 22), or who achieved stable disease for six months or more (group 2, n = 17). Tissues were immunostained for DCK and scanned images were quantified using QuPath and histoscores compared between groups (DCK nucleus p-value = 0.2102; DCK cytoplasm p-value = 0.1461; Mann-Whitney test). (**B**) Lung cancer from a partial responder, who had not been previously treated with gemcitabine and survived for 10 months while receiving NUC-1031, displayed high DCK expression. QuPath segmentation of tumour (red) and stroma (green) (left panel) or unsegmented image (right panel). Blue arrow, DCK nuclear expression. Red arrow, DCK cytoplasmic expression. (**C**) Pancreatic cancer from a patient who had relapsed on prior gemcitabine treatment but achieved a 30% reduction in tumour volume (partial response) within 3 cycles of NUC-1031 treatment, displayed low DCK expression. QuPath segmentation of tumour (red) and stroma (green) (left panel) or unsegmented image (right panel). Blue arrow, DCK negative cancer cell. Red arrow, DCK negative immune cell.

From the NUC-1031 treated Phase I cohort, 39 biopsies from 37 patients with clinical follow-up were analyzed^1^. All patients had rapidly progressing disease on study entry and had exhausted all other treatment options. Of these, two patients achieved a partial response (unconfirmed) to NUC-1031 according to RECIST 1.1 criteria, thirteen patients achieved stable disease for six months or more and twenty-two patients achieved stable disease for less than six months, or progressive disease developed within that time. Patients with progressive or stable disease for less than 6 months showed no significant difference in DCK or DCTPP1 expression by histoscore, compared to those who had stable disease for more than 6 months or had a partial response (Fig. 7A, Fig. S5). Interestingly, a lung cancer from a partial responder, who had not been previously treated with gemcitabine and survived for 10 months while receiving NUC-1031, displayed high DCK expression. A pancreatic cancer from a second patient who had relapsed on prior gemcitabine treatment but achieved a 30% reduction in tumour volume (partial response) within 3 cycles of NUC-1031 treatment, displayed low DCK expression (Fig. 7B). These data suggest that in tumours from NUC-1031 treated patients, DCK expression does not strongly correlate with disease progression.

## Discussion

Despite its widespread use as a mainstay of chemotherapy in patients with pancreatic, ovarian, lung, breast and biliary tract cancers, amongst others, the fluoropyrimidine gemcitabine achieves responses in less than 10% of patients with metastatic pancreatic cancer and has a very limited impact on overall survival due to intrinsic and acquired resistance. NUC-1031, a phosphoramidate transformation of gemcitabine, was the first anti-cancer ProTide to enter the clinic. We find that: (1) NUC-1031 and gemcitabine display important *in vitro* cytotoxicity differences; (2) the only pathway consistently selected with NUC-1031 in our CRISPR/Cas9 screen was pyrimidine metabolism, while there were no hits consistently selected with gemcitabine under our selection conditions; (3) low DCK expression in tumour biopsies from patients treated with gemcitabine correlates with a poor prognosis, but there is no such correlation in tumour biopsies from a Phase I cohort treated with NUC-1031.

Although similar in structure to gemcitabine^24^, these data demonstrate that ProTide chemistry does alter cytotoxicity and the effects of NUC-1031 are prolonged over time. These properties allowed for on-target, long-term *in vitro* selection with NUC-1031 in our genetic screening approach that consistently selected pyrimidine metabolism through the identification of DCK and DCTPP1, both of which regulate the dCMP/dCTP pool. The screening process was sufficiently sensitive to uncover DCTPP1 displaying a 1.5-fold change in sensitivity to NUC-1031. No major resistance factors to NUC-1031 were identified in this screen, since no other genes, except DCK, validated with more than a 2-fold change and the effects of DCK loss on NUC-1031 resistance were very modest compared to those of gemcitabine, with a minimal loss of NUC-1031 sensitivity in cancer cell lines.

Contrary to NUC-1031, no consistent candidates were selected from the gemcitabine screen. This may be explained by the pleiotropic effects of gemcitabine and the long exposure time to gemcitabine in our study, which generates off-target toxicity especially through the production of dFdU metabolites. There are multiple resistance-associated genes for gemcitabine, including DCK, hENT1, CDA, RRM1 and RRM2 that all converge on a common mechanism^17,34^. Previously reported gemcitabine genetic screens used shorter exposure times, when compared to our study and none of these screens selected these known resistance factors^35–37^. Since DCK, hENT1, CDA, RRM1 and RRM2 were not selected in our gemcitabine screen, although they were present in the library representation (rank position DCK >1951, hENT1 >1999, CDA >1241, RRM1 >1541, RRM2 >593, respectively), we hypothesize that there may have been adaptation responses to the gemcitabine selection pressure on the CRISPR/Cas9 library. Therefore, caution should be used when performing CRISPR/Cas9 screens, since this methodology may not uncover very strongly selected genes and may be dependent on factors such as *in vitro* drug metabolism effects.

We have used QuPath^32^ quantitative image analysis of DCK and DCTPP1 expression in tumour tissue biopsies from a Phase I patient cohort treated with NUC-1031 (ref.^1^), involving the fast and interactive training of object classifiers using machine learning techniques. The analysis of 39 biopsies, from a range of different tumours, did not show a correlation between DCK and DCTPP1 expression and efficacy of NUC-1031. Although the number of patients is small, the data suggest that NUC-1031 achieved clinical activity even in patients with low DCK expressing tumours. Further clinical studies are warranted to assess the long-term efficacy of NUC-1031 treatment in patients and monitoring dCMP/dCTP levels in NUC-1031 treated tumours may be of benefit. In conclusion, these experiments support the notion that NUC-1031 overcomes the cancer cell resistance mechanisms that limit the clinical utility of gemcitabine. Importantly, patients who had previously relapsed on gemcitabine treatment show clinical responses to NUC-1031, further confirming the potential for NUC-1031 to represent a more effective treatment option for these patients.

## Materials and Methods

### Cell culture and reagents

MiaPaCa2 and HEK293T cell lines were cultured in Dulbecco’s modified Eagle medium (DMEM) supplemented with 10% (v/v) Fetal bovine serum and 1% (v/v) Penicillin/Streptomycin. A2780 and PSN1 cell lines were cultured in RPMI 1640 supplemented with 10% (v/v) Fetal bovine serum and 1% (v/v) Penicillin/Streptomycin. Cells were routinely tested negative for Mycoplasma using the Minerva Biolabs ‘Venor GeM One Step’ PCR kit.

Human GeCKOv2 CRISPR knockout pooled library was a gift from Feng Zhang (Addgene #1000000048). Pooled lentiCRISPRv2 expression vectors containing GeCKOv2 library were provided as two half-libraries A and B, at a concentration of 50 ng/µl. plentiCRISPRv2 was a gift from Feng Zhang (Addgene #52961) and pX333 was a gift from Andrea Ventura (Addgene #64073). Gemcitabine (Sigma-Aldrich, UK) and NUC-1031 (provided by NuCana plc), were dissolved in dimethyl sulfoxide (DMSO; Sigma-Aldrich, UK) at a stock concentration of 10 mM. 2′deoxycytidine (Sigma-Aldrich, UK) was dissolved in DMSO at a stock concentration of 100 mM. Nucleofection (Amaxa) was performed on MiaPaCa2 and A2780 cells using standard methods to introduce pX333.

### Cytotoxicity assays

MiaPaCa2 and PSN1 cells were seeded in 96-well plates at a density of 500 cells per well. After 48 h, cells were incubated with culture media containing either 0.1% (v/v) DMSO or increasing concentrations of drug (gemcitabine or NUC-1031) from 5 nM to 250 nM in six experimental replicates. Cells treated with gemcitabine were washed out after 24 h and incubated with fresh media. A2780 cells were seeded in 96-well plates at a density of 750 cells per well. After 48 h, cells were incubated with culture media containing either 0.1% (v/v) DMSO or increasing concentrations of drug (gemcitabine or NUC-1031) from 5 nM to 500 nM in six experimental replicates. Cells were washed out after 2 h and incubated with fresh media. The number of drug-treated cells at d4 post-treatment was assessed by Celigo cytometer (Nexcelom Bioscience) for MiaPaCa2 and PSN1 cells or Sulforodamine B (SRB) assay for A2780 cells and normalized to the count of DMSO-treated cells (control). Cell numbers obtained from Celigo or SRB analysis, were used to generate dose response curves and to calculate the concentration to get 50% of drug effect (IC_50_) for gemcitabine and NUC-1031, using Graphpad Prism software.

### Flow cytometry

5 × 10^5^ A2780 cells were plated in 6 cm dishes and left to grow for 48 h. Cells were treated with IC_50_ of either NUC-1031 or gemcitabine or culture media containing 0.1% (v/v) DMSO for 2 h. After incubation with BrdU (10 μM) for 30 mins prior to being collected, cells were trypsinized and centrifuged at 1200 rpm for 5 min, washed with PBS and centrifuged again before being re-suspended in 1 mL of ice-cold 70% ethanol and stored at 4 °C until staining for analysis on the flow cytometer. Cells were digested with pepsin (0.4 mg/mL in 100 mM HCl – Sigma P6887) for 45 min at 37 °C after which DNA was denatured with 2N HCL/0.5% Triton X-100 for 30 min at RT and then neutralized with 0.1M sodium tetraborate pH8.5. DNA staining was performed using an anti-BrdU antibody (Beckton Dickinson, clone B44) at a 1:100 dilution in PBS/0.5% BSA/0.5% Tween20 and anti-mouse FITC (Alexa Fluor 488, Invitrogen A1101). Samples were incubated with RNAse A (Qiagen 103130) and propidium iodide. They were run on a BD FACSJazz^TM^ and data analysis was performed using FlowJo software v10.

### Lentivirus production

Both half-libraries (A and B) were used and viral particles produced independently from A and B before being combined and used to infect recipient cells. 4 × 10^6^ HEK293T cells were plated for each 10 cm dish and co-transfected the next day with 4 µg library A or B plasmids (lentiCRISPRv2), 2 µg pVSVg and 3 µg psPAX2 lentiviral packaging plasmids, using 27 µl of Trans-iT LT1 reagent (Mirus). Viral supernatant was collected 48 h and 72 h after transfection. Viral collections at 48 h and 72 h were pooled together, passed through a 0.45 µm filter in the presence of 8 µg/ml polybrene (Sigma-Aldrich) and then used to infect recipient cells. A total of 2 × 10^8^ MiaPaCa2 cells were infected with lentiviral particles containing GeCKOv2 library at a MOI of 0.3 (aiming for ~300X coverage per sgRNA). Transduced cells were then selected for 7d with 2 µg/ml puromycin (Sigma-Aldrich).

### GeCKOv2 screen for gemcitabine and NUC-1031 resistance/sensitivity

6 × 10^7^ transduced cells were collected as a baseline for sgRNA distribution at the start of the screen. Remaining MiaPaCa2 cells were divided into three conditions with a minimum of 2.6 × 10^7^ cells for each. They were either treated with DMSO as a control, 15 nM gemcitabine or 65 nM NUC-1031. Cells were maintained for four weeks and cell pellets consisting of 6 × 10^7^ cells were collected 14d and 21d after the start of the treatment. Genomic DNA was extracted from these cells using Blood and cell Midi kit (Qiagen) and sgRNA sequences isolated by PCR. A second PCR reaction was carried out on the resulting amplicons to add adapter sequences for the sequencing system and barcodes to discriminate each sample after multiplex NGS^27,28^ (Table S2). Quantification and purity of the resulting PCR products were evaluated using QubiT fluorometer, qPCR using KAPA Library Quantification Kit for Illumina platforms and Agilent 2100 Bioanalyzer system. Barcoded sgRNAs were then sequenced using the Illumina HiSeq 2500 system by Edinburgh Genomics, The University of Edinburgh.

### Sequencing data analysis

Raw sequencing reads from NGS were demultiplexed then, 5′ Illumina adapter sequences were trimmed using Cutadapt 1.3. Trimmed reads were then aligned to the reference GeCKOv2 library using Bowtie 0.12.9. The number of reads uniquely mapped to each reference sgRNA sequence was calculated and read count per sgRNA was normalized as follows: (read count per reference sequence/total of uniquely aligned reads for all sgRNAs in sample) × 10^6^. Graphic representations of sgRNA counts were generated using R Studio software. Read counts from each sample were then analyzed using the MAGeCK algorithm^29^ to rank and prioritize sgRNAs and genes affecting MiaPaCa2 cells sensitivity or resistance to gemcitabine and NUC-1031.

### Western Blotting analysis

1 × 10^6^ cells were plated onto 10 cm petri dishes and left to grow for 48 h. Cells were washed with ice-cold PBS and lysed in a lysis buffer composed of: 10 mM Tris pH 8.0, 150 mM NaCl, 1% sodium deoxycholate, 1% Nonidet P-40 (NP40), 1% Sodium dodecyl sulfate (SDS), 1 mM EDTA and supplemented with 1X protease inhibitor (AMV Roche), phosphatase inhibitor cocktail 2 and 3 (Sigma-Aldrich), 2 mM phenylmethylsulfonyl fluoride (PMSF) (SigmaAldrich). 200 µL of supplemented lysis buffer were added to each plate, then protein extracts were collected and quantified by Bicinchoninic Acid (BCA) assay using Pierce BCA protein assay kit (Thermo Scientific, UK). 30 µg of lysates were resolved by SDS Polyacrylamide Gel Electrophoresis, then transferred on a PVDF membrane. Immunoblotting was carried out at 4 °C overnight using the following antibodies: XTP3TPA/DCTPP1 (B-6) (Santa Cruz #sc-398501), DCK (Genetex **#**GTX102800), GAPDH (Sigma-Aldrich #G8795), β actin (Cell Signaling Technology #8H10D10). GAPDH and β actin were used as loading controls.

### Immunohistochemistry and machine learning analysis

Tissue microarray (TMA) samples were obtained from a cohort of patients with pancreatic cancer (Table S3). Ethical approval was granted by Scotland A REC (10/S1402/33) for the generic use of pathology archive tissue for research. Whole section slides from a pan-cancer Phase I cohort were also obtained (Table S4) where all biopsies were taken prior to NUC-1031 treatment^1^. Corresponding clinical data was also obtained from clinical records including tumour diagnosis, sex, and details on previous chemotherapy, and response if any to NUC-1031. Since ORR was not a primary focus, routine scans were conducted every 8 weeks. Patients did not receive confirmatory scans, i.e., 4 weeks after initial documentation of response, as per RECIST 1.1. All data was rendered patient non-identifiable prior to receipt. All methods were carried out in accordance with relevant guidelines and regulations and informed consent was obtained from all subjects. All experimental protocols were approved by University of St Andrews Teaching and Research Ethics Committee.

Slides were immersed three times in Xylene for 5 min and rehydrated in graded concentrations of alcohol (100, 100, 80 and 50%) for 2 min each and then rinsed in running water. Heat-induced antigen retrieval was performed in boiling Citrate buffer (10 mM, pH 6.0) at 99 °C in an automatic pressure cooker for 5 min. Endogenous peroxide activity was blocked by incubation of the slides with 3% hydrogen peroxide, for 5 min followed by a 5 min wash in 0.1% PBS-T. Serum-free block solution (DAKO, Agilent, UK) was added on the TMA for 10 min. DCTPP1 and DCK primary antibodies (XTP3TPA/DCTPP1 (B-6) (Santa Cruz #sc-398501), DCK (Genetex **#**GTX102800)), were diluted in DAKO diluent to 1:500 and 1:1500 respectively. EnVision HRP-conjugated anti-Mouse or anti-Rabbit secondary antibody were added to the appropriate TMA. DAB chromogen (DAKO, Agilent, UK) was added to each slide for 10 min. Tissues were dehydrated, cleared in Xylene and then mounted with DPX mounting medium (Sigma-Aldrich, UK) and left to dry overnight. Slides were scanned and imaged using a Leica SCN400 brightfield microscope. The analysis of stained tissues images was carried out using QuPath software. Four categories of staining intensity were then created using three different thresholds to classify cells according to their staining intensity: cells with an optical density (OD) below 0.2 were considered negative, OD between 0.2 and 0.4 were weakly positive (1+), between 0.4 and 0.6 were moderately positive (2+) and above 0.6 were strongly positive (3+). QuPath was then trained to distinguish different tissue areas and cellular types within tissue cores (including non-neoplastic cells, tumour cells, immune cells, stroma, red blood cells, necrosis), by drawing around representative areas or cells and annotating them. The set parameters were then applied to analyze all tissue cores. Each tissue core was then controlled to ensure that only tumour cells were analysed, and all areas analysed outside tumour cells were removed. QuPath calculated histoscores of each case, based on the staining intensity within tumour cells and their proportion.

### Statistical analysis

Statistical analysis of sgRNA count were performed using R studio software and MAGeCK program. All other statistical analyses were conducted using Graphpad Prism software. Mann Whitney U test was used when at least four replicates were available. P value < 0.05 was considered statistically significant.

## Supplementary information

Supplementary information
