# Supplementary material for: Genome-scale CRISPR/Cas9 screen determines factors modulating sensitivity to ProTide NUC-1031
Source: Sci Rep. 2019 May 21;9:7643. doi: 10.1038/s41598-019-44089-3 (PMC6529431; doi:10.1038/s41598-019-44089-3)
Supplement: Supplementary file 1 — Supplementary information [file 41598_2019_44089_MOESM1_ESM.pdf]

Genome-scale CRISPR/Cas9 screen determines factors modulating sensitivity to ProTide NUC-1031

Awa Sarr[1,2 §], Jennifer Bré[1§], In Hwa Um[1], Tsz Huen Chan[1], Peter Mullen[1], David J. Harrison[1], Paul A. Reynolds[1,2*]

[1]*School of Medicine, University of St Andrews, St Andrews, UK, KY16 9TF.*

[2]*Biomedical Sciences Research Complex, University of St. Andrews, St. Andrews, UK.*

[§]The authors contributed equally to this work

[*]Corresponding author

Email: par10@st-andrews.ac.uk

Supplementary Information

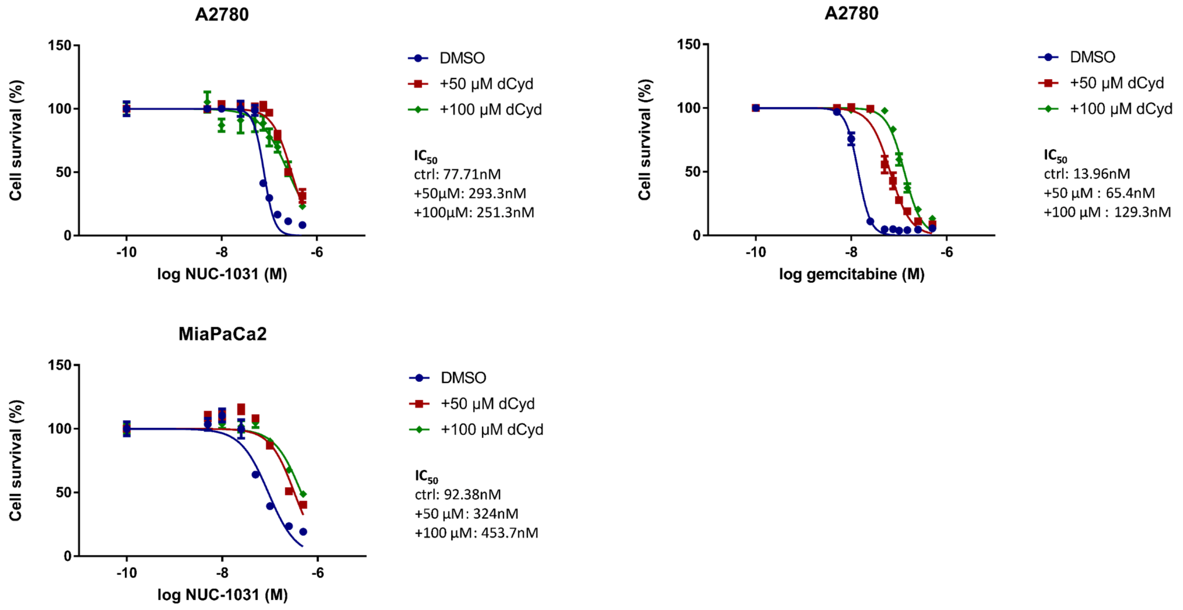

Figure S1: Dose-response curves for A2780 or MiaPaCa2 cells 4d after treatment with NUC-1031 or gemcitabine that were pre-treated when cells were plated with either DMSO, 50 µM or 100 µM of deoxycytidine (dCyd). Values represent mean +/- SEM (n=6).

A

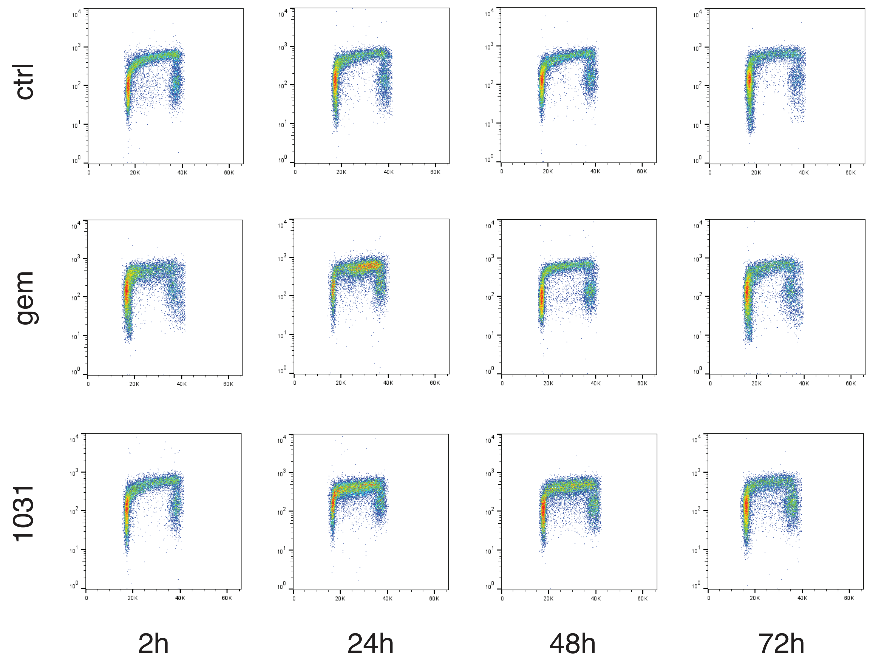

B

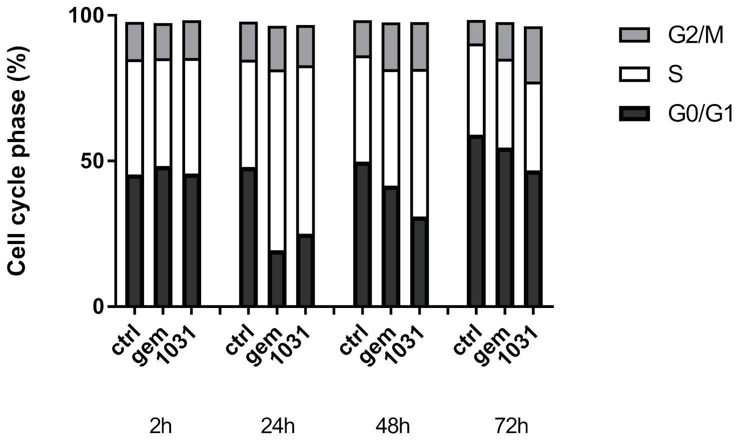

Figure S2: (A) Cell cycle analysis of A2780 cells, post treatment with IC_{50} of gemcitabine or NUC-1031 or DMSO (ctrl) for 2h. Horizontal axis, DNA content; vertical axis, BrdU incorporation. (B) Quantification of percentage of cell population in different phases of the cell cycle.

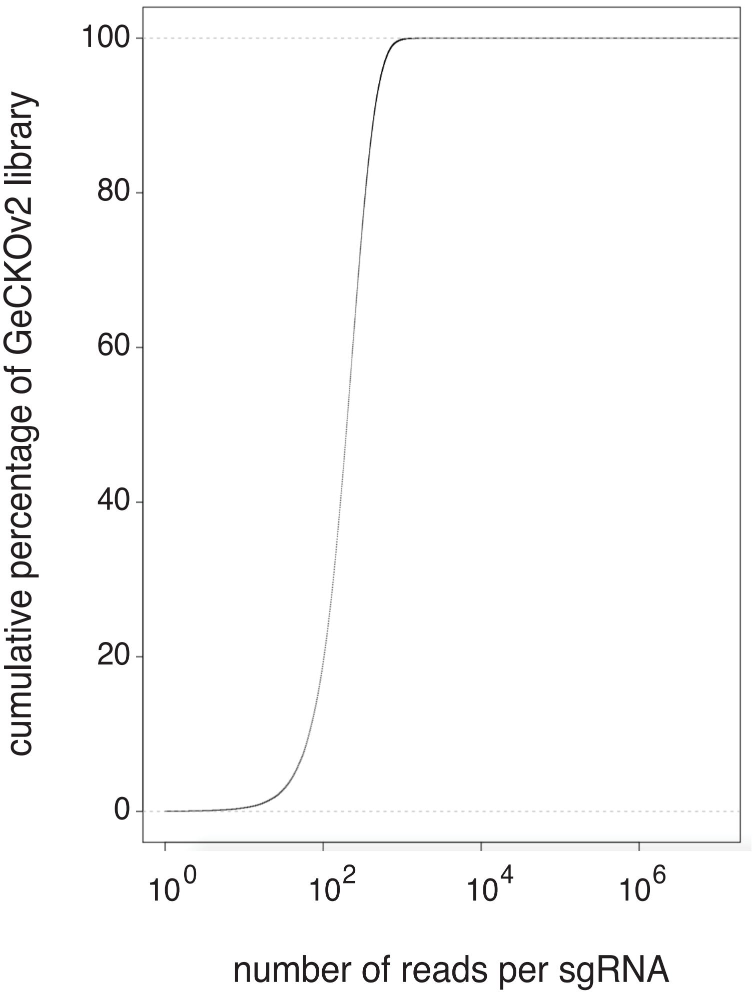

Fig. S3: Read coverage per sgRNA for GeCKOv2 library

Cumulative distribution of the number of reads per sgRNA in a single MiaPaCa2 experiment. Less than 1% of the sgRNAs are covered by less than 10 reads.

NUC-1031 d14 R1 vs R2

id	num	neg|score	neg|p-value	neg|fdr	neg|rank	neg|goodsgrna	pos|score	pos|p-value	pos|fdr	pos|rank	pos|goodsgrna
TMEM165	6	0.58133	0.278	0.488657	12442	0	2.0305E-11	2.259E-07	0.002475	1	5
DCK	6	0.04895	0.00085411	0.011843	1518	1	4.4996E-11	2.259E-07	0.002475	2	4
NonTargetingControlGuideForHuman_0731	2	0.99999	1	0.999997	21915	0	9.6566E-06	2.9366E-06	0.020509	3	2
KSR1	6	0.61455	0.32367	0.538803	13165	0	0.000024309	0.000004292	0.020509	4	1
PPIA	6	0.91423	0.82249	0.912376	19753	0	0.000032674	0.000006551	0.020509	5	3
NonTargetingControlGuideForHuman_0045	2	0.99997	0.99998	0.999997	21913	0	0.00003268	0.000006551	0.020509	6	2
ZBTB9	6	0.83278	0.65912	0.807207	17894	0	0.00003401	0.000006551	0.020509	7	5
ARMC3	6	0.96445	0.92298	0.965425	20950	0	0.000049861	0.000012876	0.031353	8	5
POLD2	6	0.29428	0.060485	0.211472	6237	0	0.000050501	0.000012876	0.031353	9	4
CA5A	6	0.63449	0.34467	0.555248	13600	0	0.00005648	0.000017846	0.036454	10	4
EEF1B2	6	0.50097	0.18961	0.386046	10735	0	0.000059526	0.000018298	0.036454	11	2
CYP3A43	6	0.18569	0.025088	0.137525	3984	1	0.00007584	0.000022364	0.0377	12	3
CYBRD1	6	0.97722	0.95256	0.980585	21287	0	0.000076187	0.000022364	0.0377	13	3
SIRPD	6	0.98992	0.97998	0.992856	21629	0	0.000099437	0.000029592	0.046322	14	5
SPATA21	6	0.96126	0.91315	0.959196	20863	0	0.00010749	0.000036821	0.053795	15	4
SAR1A	6	0.99998	0.99999	0.999997	21914	0	0.00011127	0.000041791	0.05724	16	5
TSEN54	6	0.874	0.74823	0.869393	18855	0	0.00011823	0.000045857	0.058699	17	3
IL17D	6	0.23802	0.034195	0.146755	5058	1	0.00012154	0.000049019	0.058699	18	1
PPAT	6	0.85732	0.71418	0.845771	18491	0	0.00012372	0.000051278	0.058699	19	4
S100A8	6	0.99807	0.99725	0.999854	21853	0	0.00012772	0.000055344	0.058699	20	5
NACC2	6	0.40426	0.10911	0.277176	8590	0	0.00012954	0.000056248	0.058699	21	3
ING3	6	0.98337	0.96558	0.986746	21444	0	0.00013744	0.000071609	0.071332	22	4
GLDC	6	0.92535	0.84484	0.926104	19986	0	0.00015321	0.000085614	0.081576	23	4
TRPC5	6	0.59048	0.2874	0.498022	12641	0	0.00017015	0.00008968	0.081889	24	1
NonTargetingControlGuideForHuman_0700	2	0.99981	0.99989	0.999997	21910	0	0.00018697	0.000095102	0.082445	25	2
JRKL	6	0.98951	0.97945	0.992686	21620	0	0.00019618	0.000097813	0.082445	26	4
TMEM131	6	0.053139	0.0010122	0.013526	1589	1	0.00021876	0.00010278	0.083425	27	1
ABI3	6	0.40095	0.10883	0.277176	8528	0	0.00023123	0.0001082	0.084689	28	3
SYNCRIP	6	0.012911	0.00023967	0.007105	539	2	0.00027577	0.00012492	0.090135	29	3
DCTPP1	6	0.99856	0.99805	0.99987	21875	0	0.00028982	0.00013305	0.090135	30	4

id	num	neg|score	neg|p-value	neg|fdr	neg|rank	neg|goodsgrna	pos|score	pos|p-value	pos|fdr	pos|rank	pos|goodsgrna
TMEM165	6	0.84243	0.72131	0.864273	18289	0	1.0747E-06	2.259E-07	0.00495	1	5
GNPNAT1	6	0.75786	0.59729	0.797353	16335	0	8.5927E-06	6.7769E-07	0.007426	2	3
PHF19	6	0.93591	0.89753	0.963369	20413	0	0.00001288	2.0331E-06	0.012871	3	3
SLC25A46	6	0.97729	0.96917	0.993792	21372	0	0.000019108	2.9366E-06	0.012871	4	3
PMS1	6	0.99694	0.99756	0.999995	21840	0	0.000020289	2.9366E-06	0.012871	5	3
DCK	6	0.92039	0.87188	0.951818	20070	0	0.000024309	3.8402E-06	0.014026	6	2
TNKS2	6	0.99992	0.99998	0.999995	21914	0	0.000029022	5.6474E-06	0.01547	7	5
GABRG1	6	0.99997	1	0.999995	21915	0	0.000029453	5.6474E-06	0.01547	8	6
ARHGAP40	6	0.25139	0.12072	0.361844	7306	1	0.000057141	0.00001378	0.027921	9	2
DPH5	6	0.84419	0.72706	0.868948	18324	0	0.000064603	0.000014683	0.027921	10	5
GAPT	6	0.79535	0.6493	0.825413	17189	0	0.000072925	0.000017394	0.027921	11	3
hsa-mir-454	4	0.98841	0.98653	0.999284	21631	0	0.000080093	0.000017846	0.027921	12	3
LOC154872	6	0.99911	0.99954	0.999995	21894	0	0.00011003	0.00002146	0.027921	13	5
OSBPL8	6	0.69616	0.51303	0.749588	14989	0	0.00012154	0.000022364	0.027921	14	1
NonTargetingControlGuideForHuman_0814	2	0.99987	0.99997	0.999995	21912	0	0.0001275	0.000022815	0.027921	15	2
BRAP	6	0.055158	0.016374	0.141117	2523	1	0.00013373	0.000022815	0.027921	16	4
hsa-mir-301b	4	0.092874	0.029504	0.175439	3545	2	0.00014585	0.000024171	0.027921	17	1
COQ4	6	0.8739	0.78682	0.907025	19002	0	0.00017015	0.00002643	0.027921	18	3
AGO3	6	0.88478	0.80996	0.920704	19263	0	0.00017023	0.00002643	0.027921	19	4
PRR23C	6	0.058813	0.017362	0.143997	2631	2	0.00017094	0.000027785	0.027921	20	2
SMPX	6	0.94857	0.92118	0.974653	20704	0	0.00018493	0.000029592	0.027921	21	5
ZDHHC20	6	0.96751	0.9536	0.988679	21132	0	0.00018604	0.000029592	0.027921	22	4
CMC4	6	0.80431	0.65457	0.825413	17375	0	0.00020411	0.000031399	0.027921	23	3
IQCG	6	0.97849	0.97147	0.994221	21401	0	0.00020673	0.000031851	0.027921	24	4
CALCR	6	0.9996	0.99987	0.999995	21905	0	0.0002069	0.000031851	0.027921	25	5
LIPA	6	0.38165	0.17166	0.414814	9060	1	0.00025583	0.000036369	0.030655	26	2
CCDC42B	6	0.0074639	0.00093317	0.038	527	1	0.00026737	0.00003908	0.03172	27	3
HEATR5B	6	0.8926	0.81772	0.920704	19445	0	0.00027464	0.000043598	0.034123	28	5
LPP	6	0.97952	0.97254	0.994785	21422	0	0.00029316	0.000048116	0.036361	29	5
ITGA7	6	0.94415	0.91259	0.97089	20599	0	0.0003018	0.000049923	0.036469	30	5

NUC-1031 d21 R1 vs R2

id	num	neg|score	neg|p-value	neg|fdr	neg|rank	neg|goodsgrna	pos|score	pos|p-value	pos|fdr	pos|rank	pos|goodsgrna
TMEM165	6	0.9997	0.99938	0.999998	21899	0	2.7363E-09	2.259E-07	0.00165	1	5
DCK	6	0.12453	0.074292	0.265079	6123	1	2.8997E-08	2.259E-07	0.00165	2	4
NonTargetingControlGuideForHuman_0476	2	1	1	0.999998	21915	0	1.3521E-06	2.259E-07	0.00165	3	2
OIP5	6	0.35688	0.25973	0.486271	11695	1	3.0966E-06	1.1295E-06	0.006188	4	4
KRT5	6	0.73425	0.59382	0.76262	17053	1	9.1127E-06	5.1956E-06	0.018977	5	4
PPIA	6	0.90714	0.79332	0.887981	19566	1	9.5352E-06	5.1956E-06	0.018977	6	2
PPAT	6	0.54415	0.4266	0.644342	14489	1	0.000022438	0.000024623	0.061056	7	3
TRIM54	6	0.95293	0.8878	0.941229	20651	0	0.000023569	0.000024623	0.061056	8	4
WFDC9	6	0.98165	0.94854	0.972046	21383	0	0.000024309	0.000025074	0.061056	9	1
NonTargetingControlGuideForHuman_0045	2	0.99997	0.99995	0.999998	21913	0	0.000033519	0.000028237	0.061881	10	2
NonTargetingControlGuideForHuman_0731	2	0.99993	0.9999	0.999998	21912	0	0.000068111	0.000042242	0.078218	11	2
XPO1	6	0.19351	0.12752	0.349893	7941	3	0.000072925	0.000049019	0.078218	12	1
NonTargetingControlGuideForHuman_0409	2	0.99992	0.99988	0.999998	21911	0	0.000075524	0.000049923	0.078218	13	2
NonTargetingControlGuideForHuman_0031	2	0.99992	0.99988	0.999998	21910	0	0.000076089	0.000050601	0.078218	14	2
POMP	6	0.80309	0.64726	0.794766	17841	1	0.000086578	0.000053537	0.078218	15	3
NonTargetingControlGuideForHuman_0807	2	0.9999	0.99982	0.999998	21909	0	0.00010366	0.000066187	0.085891	16	2
DDO	6	0.85953	0.71708	0.840993	18677	1	0.00010681	0.00006935	0.085891	17	3
NonTargetingControlGuideForHuman_0939	2	0.99988	0.99977	0.999998	21907	0	0.00012153	0.000074771	0.085891	18	2
CCDC25	6	0.22808	0.15268	0.379557	8782	2	0.00012154	0.000074771	0.085891	19	1
MDGA2	6	0.37437	0.2701	0.49455	11948	2	0.00013196	0.000078386	0.085891	20	2
EEF1B2	6	0.12299	0.074122	0.265079	6089	2	0.00015896	0.000091939	0.091046	21	2
TSEN54	6	0.71749	0.5808	0.752833	16905	1	0.00016886	0.000095102	0.091046	22	3
FAM53A	6	0.98374	0.95338	0.974369	21443	0	0.00017015	0.000095554	0.091046	23	2
NonTargetingControlGuideForHuman_0424	2	0.99981	0.99962	0.999998	21903	0	0.00018653	0.00010956	0.096436	24	2
MRPL3	6	0.51664	0.3919	0.609929	14078	2	0.00019015	0.00011001	0.096436	25	2
NonTargetingControlGuideForHuman_0203	2	0.99979	0.99955	0.999998	21902	0	0.0002112	0.00012537	0.105674	26	2
EFNA5	6	0.92421	0.82832	0.908605	19972	0	0.00021876	0.00013125	0.106527	27	1
NACC2	6	0.73565	0.59416	0.76262	17066	1	0.00024894	0.00014886	0.109709	28	3
WDPCP	6	0.99201	0.9772	0.989021	21652	0	0.0002633	0.00015338	0.109709	29	3
RNF220	6	0.30274	0.21622	0.44371	10664	2	0.00026737	0.00015474	0.109709	30	1

id	num	neg|score	neg|p-value	neg|fdr	neg|rank	neg|goodsgrna	pos|score	pos|p-value	pos|fdr	pos|rank	pos|goodsgrna
SAFB	6	0.35868	0.0096803	0.026071	7765	0	3.8873E-06	2.259E-07	0.000707	1	3
NonTargetingControlGuideForHuman_0815	2	1	1	1	21915	0	4.5935E-06	2.259E-07	0.000707	2	2
IGDCC3	6	0.78174	0.082549	0.102282	17685	0	0.000016217	2.259E-07	0.000707	3	2
hsa-mir-4311	4	0.77611	0.079863	0.099875	17520	0	0.000019456	2.259E-07	0.000707	4	2
CORO1A	6	0.46781	0.018801	0.038268	10176	0	0.000024309	2.259E-07	0.000707	5	1
ANAPC7	6	0.76076	0.070159	0.090157	17054	0	0.00003287	2.259E-07	0.000707	6	3
GLT8D1	6	0.24532	0.0036742	0.013893	5337	1	0.000034823	2.259E-07	0.000707	7	4
NonTargetingControlGuideForHuman_0345	2	0.99994	1	1	21914	0	0.000056605	4.7438E-06	0.011251	8	2
ARFGAP3	6	0.8851	0.13877	0.149749	20304	0	0.000062945	5.1956E-06	0.011251	9	3
CCDC71L	6	0.92231	0.1756	0.18261	21073	0	0.000072925	0.000011972	0.011251	10	1
PALMD	6	0.082891	0.0028131	0.013893	2093	1	0.000073103	0.000011972	0.011251	11	3
ZNF689	6	0.64827	0.042397	0.065021	14247	0	0.00012154	0.000012424	0.011251	12	1
MRPL55	6	0.58976	0.033607	0.056713	12909	0	0.00014665	0.000012876	0.011251	13	2
GPR75	6	0.33081	0.0094914	0.026071	7176	0	0.0001641	0.000013328	0.011251	14	2
HPRT1	6	0.56526	0.033134	0.056713	12362	0	0.0001644	0.000013328	0.011251	15	3
MROH1	6	0.65896	0.043269	0.065053	14505	0	0.00017015	0.000013328	0.011251	16	2
TMED3	6	0.67372	0.048964	0.071589	14859	0	0.00018631	0.000014683	0.011251	17	3
ZMYM6	6	0.67552	0.049054	0.071589	14899	0	0.00018778	0.000015135	0.011251	18	3
KRTAP9-1	6	0.57868	0.033479	0.056713	12667	0	0.00019882	0.000015135	0.011251	19	2
PCDH11Y	6	0.98795	0.88805	0.888621	21901	0	0.00021876	0.000015135	0.011251	20	1
GCM2	6	0.94349	0.23194	0.237049	21443	0	0.00023779	0.000015135	0.011251	21	2
ZIK1	6	0.0040285	4.7438E-06	0.000252	212	1	0.00023923	0.000015135	0.011251	22	2
hsa-mir-4499	4	0.202	0.0034176	0.013893	4430	0	0.00024307	0.000015135	0.011251	23	1
SAP130	6	0.46969	0.018806	0.038268	10220	0	0.00024991	0.000015135	0.011251	24	2
MT1G	6	0.86943	0.1325	0.145296	19962	0	0.00026737	0.000015587	0.011251	25	1
COLEC12	6	0.84667	0.11933	0.134434	19416	0	0.00028753	0.000015587	0.011251	26	2
SORCS1	6	0.92654	0.18398	0.190484	21165	0	0.0003046	0.000015587	0.011251	27	2
SLC18B1	6	0.83258	0.11398	0.131048	19056	0	0.00031598	0.000015587	0.011251	28	1
RFC1	6	0.95501	0.26878	0.272578	21610	0	0.00031994	0.000015587	0.011251	29	2
TACC1	6	0.70272	0.053458	0.075412	15527	0	0.00035756	0.000016039	0.011251	30	3

Gemcitabine d14 R1 vs R2

id	num	neg|score	neg|p-value	neg|fdr	neg|rank	neg|goodsgrna	pos|score	pos|p-value	pos|fdr	pos|rank	pos|goodsgrna
HEATR5B	6	0.98782	0.98748	0.998792	21663	0	9.1206E-06	2.259E-07	0.002475	1	5
hsa-mir-4693	2	0.99999	1	1	21915	0	0.000012567	2.259E-07	0.002475	2	2
hsa-mir-663b	4	0.99712	0.99832	1	21843	0	0.000015724	6.7769E-07	0.003536	3	3
SEL1L3	6	0.22173	0.10444	0.356762	6343	1	0.000019593	1.1295E-06	0.003536	4	4
MICA	6	0.18638	0.084602	0.314942	5873	1	0.000024309	1.1295E-06	0.003536	5	2
HMG20A	6	0.37638	0.18682	0.468539	8642	1	0.000028066	1.1295E-06	0.003536	6	5
GADD45B	6	0.54724	0.3411	0.629554	11779	1	0.000029046	1.1295E-06	0.003536	7	4
RPL41	4	0.49762	0.28983	0.585966	10810	0	0.000048617	2.0331E-06	0.005569	8	2
METTL6	6	0.98443	0.98242	0.997242	21589	0	0.000059513	2.4848E-06	0.006051	9	5
PCDH18	6	0.98342	0.98081	0.996633	21565	0	0.000064555	2.9366E-06	0.006436	10	4
hsa-mir-4672	4	0.58026	0.37324	0.656137	12465	0	0.000080951	3.8402E-06	0.007235	11	3
DAPK2	6	0.67232	0.48813	0.735445	14521	0	0.00009251	0.000004292	0.007235	12	3
STAT5B	6	0.48559	0.27156	0.560661	10562	1	0.000092962	0.000004292	0.007235	13	3
LPHN1	6	0.11601	0.036747	0.199618	4003	1	0.00010729	4.7438E-06	0.007426	14	4
EIF4G3	6	0.14926	0.053827	0.238987	4905	1	0.0001371	7.0028E-06	0.009592	15	4
FRA10AC1	5	0.99986	0.99998	1	21912	0	0.00013717	7.0028E-06	0.009592	16	5
hsa-mir-3145	3	0.59739	0.39499	0.673378	12830	0	0.0001377	7.4545E-06	0.00961	17	2
GCC1	6	0.53144	0.32068	0.612138	11455	1	0.00014897	7.9063E-06	0.009626	18	4
ATP6V0B	6	0.30662	0.14641	0.425369	7537	1	0.00017015	8.8099E-06	0.009653	19	2
hsa-mir-873	4	0.20708	0.095469	0.33893	6150	1	0.00017177	8.8099E-06	0.009653	20	3
FRMD3	6	0.29846	0.13484	0.397839	7424	1	0.00017835	0.000010165	0.01052	21	5
NACAD	6	0.54547	0.34075	0.629554	11740	1	0.0001936	0.000011069	0.01052	22	3
RSPO4	6	0.12392	0.039211	0.203872	4202	1	0.0001962	0.000011069	0.01052	23	3
PTBP2	6	0.6706	0.48753	0.735445	14494	0	0.00020208	0.000011521	0.01052	24	5
hsa-mir-95	4	0.96476	0.95273	0.987656	21134	0	0.0002304	0.000013328	0.010726	25	3
DOLPP1	6	0.98763	0.98706	0.998692	21658	0	0.00023445	0.000013328	0.010726	26	3
TMEM60	6	0.4384	0.2328	0.526294	9682	1	0.00024307	0.00001378	0.010726	27	2
POLI	6	0.082574	0.021805	0.15215	3093	1	0.00024307	0.00001378	0.010726	28	4
hsa-mir-942	4	0.6003	0.39737	0.673378	12900	0	0.00026287	0.000014683	0.010726	29	2
OR4D5	6	0.43298	0.22348	0.510225	9586	1	0.00026521	0.000014683	0.010726	30	4

id	num	neg|score	neg|p-value	neg|fdr	neg|rank	neg|goodsgrna	pos|score	pos|p-value	pos|fdr	pos|rank	pos|goodsgrna
hsa-mir-3143	4	1	1	1	21915	0	2.0043E-06	2.259E-07	0.00099	1	4
NAA50	6	1	1	1	21914	0	3.4457E-06	2.259E-07	0.00099	2	6
PPP1R32	6	0.1958	0.10963	0.386151	6203	1	8.8399E-06	2.259E-07	0.00099	3	4
DBF4	5	0.67889	0.50488	0.753855	14672	0	0.000017508	2.259E-07	0.00099	4	3
CLEC19A	6	0.018478	0.0040049	0.081417	1076	2	0.000024309	2.259E-07	0.00099	5	1
hsa-mir-1273h	4	0.73923	0.59503	0.811744	16033	0	0.000034989	6.7769E-07	0.002475	6	2
DPF1	6	0.99996	1	1	21913	0	0.00003929	1.1295E-06	0.003094	7	6
NEURL1B	6	0.99996	1	1	21912	0	0.000040582	1.1295E-06	0.003094	8	6
CLTA	6	0.88043	0.81506	0.932703	19147	0	0.000043442	1.5813E-06	0.003465	9	3
GCFC2	6	0.23082	0.1387	0.425188	7111	1	0.000045919	1.5813E-06	0.003465	10	3
hsa-mir-4711	4	0.99995	1	1	21911	0	0.000052531	2.4848E-06	0.003484	11	4
IWS1	6	0.99995	1	1	21910	0	0.00005493	2.4848E-06	0.003484	12	6
NADK2	6	0.046126	0.017752	0.17244	2256	3	0.000055734	2.4848E-06	0.003484	13	2
MILR1	6	0.13894	0.065769	0.303048	4751	2	0.000058377	2.9366E-06	0.003484	14	3
AGO2	6	0.63648	0.45666	0.729215	13710	0	0.000068256	2.9366E-06	0.003484	15	3
C1orf141	6	0.53098	0.33123	0.622947	11572	1	0.000072925	3.3884E-06	0.003484	16	2
GPC6	6	0.87802	0.81062	0.929626	19098	0	0.000090719	3.3884E-06	0.003484	17	2
NonTargetingControlGuideForHuman_0555	2	0.99991	0.99999	1	21908	0	0.00009447	3.3884E-06	0.003484	18	2
CACTIN	6	0.90657	0.85466	0.948541	19744	0	0.000096142	3.3884E-06	0.003484	19	2
hsa-mir-548h-5	3	0.96877	0.96092	0.993987	21186	0	0.000098221	3.3884E-06	0.003484	20	2
ZNF439	6	0.90776	0.85595	0.948583	19773	0	0.00010585	3.8402E-06	0.003484	21	4
HAUS7	6	0.72332	0.56859	0.79571	15657	0	0.00012154	3.8402E-06	0.003484	22	1
PYROXD1	6	0.1497	0.071979	0.312982	5022	2	0.0001256	3.8402E-06	0.003484	23	3
FXN	6	0.96717	0.95942	0.993846	21140	0	0.00013683	3.8402E-06	0.003484	24	4
NonTargetingControlGuideForHuman_0280	2	0.99986	0.99998	1	21907	0	0.00014198	0.000004292	0.003484	25	2
SERPINB7	6	0.57687	0.37768	0.662482	12440	1	0.00014829	0.000004292	0.003484	26	4
TREX2	6	0.93809	0.91295	0.977972	20458	0	0.00015111	0.000004292	0.003484	27	5
HILPDA	6	0.78784	0.66799	0.856729	17074	0	0.00016886	4.7438E-06	0.003713	28	3
NonTargetingControlGuideForHuman_0288	2	0.99983	0.99997	1	21906	0	0.00016945	4.9697E-06	0.003756	29	2
TRAF3IP1	6	0.47752	0.28738	0.583015	10667	1	0.00017015	5.6474E-06	0.004125	30	3

Gemcitabine d21 R1 vs R2

id	num	neg|score	neg|p-value	neg|fdr	neg|rank	neg|goodsgrna	pos|score	pos|p-value	pos|fdr	pos|rank	pos|goodsgrna
DAZL	6	0.021702	0.0076508	0.135992	1232	2	7.2437E-06	6.7769E-07	0.011551	1	4
USP48	6	0.99998	1	0.999998	21915	0	0.000018048	1.1295E-06	0.011551	2	6
ADAMTS18	6	0.4888	0.337	0.679553	10855	1	0.000024309	1.5813E-06	0.011551	3	1
CRYGC	6	0.25423	0.16722	0.498466	7339	1	0.000047047	5.1956E-06	0.023652	4	4
MILR1	6	0.31091	0.19898	0.532885	8091	1	0.000051617	5.6474E-06	0.023652	5	4
HS3ST4	6	0.94325	0.94455	0.999998	20609	0	0.000059898	8.8099E-06	0.023652	6	4
NonTargetingControlGuideForHuman_0555	2	0.99994	0.99999	0.999998	21914	0	0.00006146	8.8099E-06	0.023652	7	2
FCHO1	6	0.84802	0.81427	0.966247	18467	0	0.000072925	0.000011521	0.023652	8	2
FKRP	6	0.99993	0.99999	0.999998	21913	0	0.000073987	0.000011521	0.023652	9	6
TBX21	6	0.99726	0.99868	0.999998	21844	0	0.00008545	0.000014683	0.023652	10	5
PTGER2	6	0.54438	0.40118	0.740287	11846	1	0.00011414	0.000016942	0.023652	11	4
SLC30A6	6	0.015913	0.0047418	0.106253	976	2	0.00012154	0.000017394	0.023652	12	3
GRB7	6	0.98605	0.99012	0.999998	21588	0	0.00012466	0.000017394	0.023652	13	4
IL17RE	6	0.023611	0.0083927	0.138186	1325	1	0.0001321	0.000017846	0.023652	14	3
PSD4	6	0.98939	0.992	0.999998	21658	0	0.00013491	0.000017846	0.023652	15	5
FRAT2	6	0.9241	0.92273	0.998461	20207	0	0.00013549	0.000018298	0.023652	16	5
FGF19	6	0.071044	0.03352	0.246011	2986	1	0.00014985	0.000021008	0.023652	17	5
HS1BP3	6	0.70188	0.59747	0.860049	15207	0	0.00015388	0.000021008	0.023652	18	4
COPZ2	6	0.15882	0.092264	0.377499	5324	1	0.00016524	0.000023267	0.023652	19	5
SYT4	6	0.02263	0.0079246	0.136424	1273	2	0.00017175	0.000024623	0.023652	20	2
SYNPO	6	0.60777	0.46229	0.7692	13109	1	0.0001748	0.000024623	0.023652	21	4
hsa-mir-1250	4	0.021986	0.0078234	0.136424	1241	1	0.00017825	0.000025074	0.023652	22	1
PHLDA2	6	0.99499	0.99722	0.999998	21787	0	0.00017832	0.000025074	0.023652	23	5
CCDC25	6	0.42054	0.26862	0.605615	9709	1	0.00019446	0.000027785	0.023652	24	1
KRTAP9-9	6	0.014233	0.0042746	0.103739	880	1	0.00019446	0.000027785	0.023652	25	2
RABGGTA	6	0.93401	0.93065	0.998979	20412	0	0.00020181	0.000028689	0.023652	26	5
CEBPA	6	0.96984	0.97529	0.999998	21201	0	0.00020603	0.00002914	0.023652	27	5
FOXA2	6	0.99985	0.99997	0.999998	21912	0	0.00023451	0.000031399	0.023852	28	4
CELSR3	6	0.88785	0.87117	0.983851	19404	0	0.0002385	0.000032303	0.023852	29	5
SHD	6	0.86238	0.83848	0.974875	18799	0	0.00023906	0.000032755	0.023852	30	5

id	num	neg|score	neg|p-value	neg|fdr	neg|rank	neg|goodsgrna	pos|score	pos|p-value	pos|fdr	pos|rank	pos|goodsgrna
TSC2	6	0.92331	0.76868	0.842135	20002	0	2.2475E-07	2.259E-07	0.00165	1	5
hsa-mir-6870	4	1	1	1	21915	0	8.741E-07	2.259E-07	0.00165	2	4
HIF1A	6	0.24911	0.050152	0.148648	7386	2	5.0272E-06	2.259E-07	0.00165	3	3
TSC1	6	0.95952	0.86872	0.913032	20851	0	6.7438E-06	1.1295E-06	0.006188	4	4
AMPD2	6	0.88904	0.70429	0.805509	19144	0	0.000015134	0.000004292	0.018812	5	4
BSND	6	0.93047	0.78447	0.852185	20165	0	0.000024309	9.7135E-06	0.035479	6	3
hsa-mir-1265	4	0.99997	0.99999	1	21914	0	0.000029948	0.000012876	0.040311	7	4
WIPF1	6	0.048491	0.0022479	0.020612	2355	2	0.000045526	0.000028237	0.069598	8	3
APCDD1L	6	0.99995	0.99998	1	21913	0	0.000047177	0.000028689	0.069598	9	6
hsa-mir-145	4	0.93994	0.80981	0.869607	20407	0	0.000050648	0.000031851	0.069598	10	3
hsa-mir-6133	4	0.99994	0.99997	1	21912	0	0.000059131	0.000038628	0.069598	11	4
hsa-mir-16-2	4	0.99994	0.99996	1	21911	0	0.000064018	0.00004405	0.069598	12	4
hsa-mir-6073	4	0.80911	0.51351	0.651154	17263	0	0.00006545	0.000044501	0.069598	13	2
hsa-mir-4443	4	0.82868	0.56564	0.699322	17712	0	0.000066705	0.000045857	0.069598	14	3
NLGN4X	6	0.25515	0.053712	0.15511	7515	1	0.000072925	0.000049923	0.069598	15	2
C10orf128	6	0.11525	0.0068575	0.0358	4177	2	0.000079475	0.000053537	0.069598	16	3
hsa-mir-3659	4	0.99992	0.99995	1	21910	0	0.000080936	0.000053989	0.069598	17	4
KRTAP9-1	6	0.99972	0.99969	1	21899	0	0.00011391	0.000084259	0.08995	18	5
CCDC71L	6	0.23358	0.040674	0.12748	6986	2	0.00012154	0.000092843	0.08995	19	3
hsa-mir-16-1	4	0.45862	0.11641	0.248132	10155	1	0.00012877	0.00010323	0.08995	20	2
MPP7	6	0.96185	0.87946	0.922176	20900	0	0.00013629	0.00011588	0.08995	21	4
hsa-mir-6774	4	0.99986	0.9999	1	21909	0	0.00014462	0.00012131	0.08995	22	4
hsa-mir-4314	4	0.97033	0.90246	0.934706	21158	0	0.00014585	0.00012266	0.08995	23	1
PDE7B	6	0.61637	0.23163	0.391657	12958	1	0.00014665	0.00012311	0.08995	24	4
hsa-mir-5704	4	0.99985	0.9999	1	21908	0	0.00014681	0.00012311	0.08995	25	4
hsa-mir-504	4	0.63547	0.25672	0.418922	13354	0	0.0001497	0.00012763	0.08995	26	3
hsa-mir-126	4	0.97692	0.92152	0.947188	21320	0	0.0001668	0.00013983	0.08995	27	2
CORO1A	6	0.50082	0.1341	0.270915	10847	1	0.00017015	0.00014028	0.08995	28	3
UTRN	6	0.62267	0.24298	0.406297	13077	1	0.00019015	0.0001457	0.08995	29	3
HES2	6	0.72714	0.38207	0.544706	15367	0	0.00019273	0.0001457	0.08995	30	3

Figure S4: Comparison of MAGeCK analysis gene ranking from independent library transduction replicates with NUC-1031 or gemcitabine at d14 or d21.

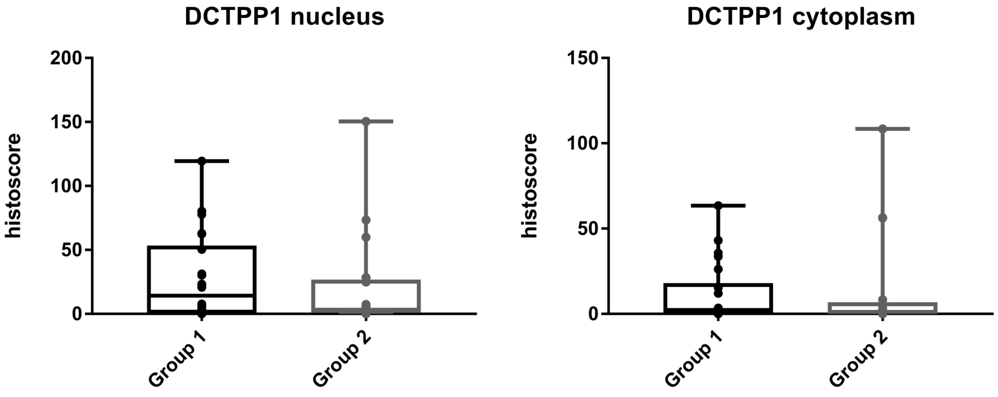

Figure S5: Patients were stable for less than six months, or progressive disease developed within that time (group 1), or were stable for six months or more (group 2). Tissues were immunostained for DCTPP1 and scanned images were quantified using QuPath. Histoscores for DCTPP1 expression (nucleus or cytoplasm) for group 1 vs group 2 are presented (nucleus group 1 vs group 2 p=0.7054, cytoplasm group 1 vs group 2 p=0.6679; Mann Whitney test).

MAGeCK analysis: gene ranking

Top 30 hits Acelarin d14

id	num	neg|score	neg|p-value	neg|fdr	neg|rank	neg|goodsgrna	pos|score	pos|p-value	pos|fdr	pos|rank	pos|goodsgrna
TMEM165	6	0.89556	0.7684	0.870669	19323	0	1.70E-16	2.26E-07	0.002475	1	5
DCK	6	0.56879	0.27278	0.492317	12114	0	1.21E-10	2.26E-07	0.002475	2	4
PYCR2	6	0.99302	0.98538	0.995277	21697	0	8.59E-06	7.00E-06	0.051155	3	2
DCTPP1	6	0.9554	0.89913	0.950441	20729	0	2.43E-05	3.23E-05	0.149615	4	5
SYNCRIP	6	0.12341	0.033067	0.169749	4165	2	3.03E-05	4.22E-05	0.149615	5	3
NonTargetingControlGuideForHuman_0731	2	0.99997	0.99998	0.999985	21915	0	3.17E-05	4.36E-05	0.149615	6	2
RASSF7	6	0.38171	0.1424	0.373018	8351	1	3.45E-05	4.99E-05	0.149615	7	3
NACC2	6	0.39357	0.15247	0.388383	8564	1	4.04E-05	5.62E-05	0.149615	8	3
NonTargetingControlGuideForHuman_0040	2	0.99996	0.99998	0.999985	21914	0	4.31E-05	6.14E-05	0.149615	9	2
COQ4	6	0.72927	0.48821	0.681733	15613	0	6.44E-05	0.00010233	0.168542	10	2
NonTargetingControlGuideForHuman_0737	2	0.99994	0.99996	0.999985	21913	0	6.48E-05	0.00010233	0.168542	11	2
LOC100288524	6	0.037069	0.0037808	0.047873	1723	2	6.70E-05	0.00011814	0.168542	12	3
TSPAN12	6	0.061216	0.011213	0.100332	2439	1	8.26E-05	0.00014435	0.168542	13	2
DCAF4	6	0.99166	0.98144	0.992835	21663	0	9.36E-05	0.000157	0.168542	14	5
NonTargetingControlGuideForHuman_0075	2	0.99991	0.99992	0.999985	21912	0	9.46E-05	0.00015926	0.168542	15	2
CA5A	6	0.80952	0.60412	0.759572	17409	0	9.48E-05	0.00015926	0.168542	16	4
MB	6	0.31836	0.095462	0.284932	7323	1	0.00010169	0.00017055	0.168542	17	5
NonTargetingControlGuideForHuman_0341	2	0.99989	0.9999	0.999985	21911	0	0.00010952	0.00017552	0.168542	18	2
ING3	6	0.99466	0.98926	0.99745	21735	0	0.00012134	0.00019314	0.168542	19	4
PTOV1	6	0.016441	0.0015544	0.035042	933	1	0.00012154	0.00019314	0.168542	20	4
SKOR1	6	0.057705	0.0084781	0.079033	2333	1	0.00013155	0.00020489	0.168542	21	4
NonTargetingControlGuideForHuman_0124	2	0.99987	0.99987	0.999985	21910	0	0.0001338	0.0002076	0.168542	22	2
ZNF583	6	0.35839	0.1198	0.329225	7963	1	0.00013629	0.00020805	0.168542	23	3
COPS7A	6	0.98104	0.95755	0.980509	21394	0	0.00013741	0.0002094	0.168542	24	4
POLA2	6	0.80588	0.5957	0.75335	17326	0	0.00013826	0.00021121	0.168542	25	4
ATXN2L	6	0.90774	0.79438	0.886342	19634	0	0.00014903	0.00021889	0.168542	26	4
ITIH4	6	0.34886	0.11698	0.326132	7792	1	0.00015444	0.00022612	0.168542	27	3
NonTargetingControlGuideForHuman_0397	2	0.99984	0.99984	0.999985	21907	0	0.00015521	0.00022657	0.168542	28	2
VPS54	6	0.40552	0.15962	0.396167	8781	1	0.0001669	0.0002469	0.168542	29	3

MAGeCK analysis: gene ranking
Top 30 hits Acelarin d21

id	num	neg|score	neg|p-value	neg|fdr	neg|rank	neg|goodsgrna	pos|score	pos|p-value	pos|fdr	pos|rank	pos|goodsgrna
TMEM165	6	0.98768	0.927	0.942479	21555	0	2.18E-12	2.26E-07	0.00495	1	5
DCK	6	0.76661	0.38221	0.50634	16535	0	2.80E-07	2.48E-06	0.027228	2	5
NonTargetingControlGuideForHuman_0397	2	1	0.99997	0.999974	21915	0	3.70E-06	4.27E-05	0.311881	3	2
SIRPD	6	0.99413	0.96151	0.970096	21721	0	9.06E-06	0.00012808	0.589109	4	5
TSPAN12	6	0.015437	0.00040955	0.009497	900	2	9.73E-06	0.00013441	0.589109	5	2
HCN3	6	0.51095	0.16972	0.33742	10993	1	1.50E-05	0.00019224	0.702145	6	5
POMP	6	0.6116	0.23302	0.388634	13124	0	1.92E-05	0.0002582	0.808345	7	4
NonTargetingControlGuideForHuman_0203	2	0.99996	0.99972	0.999764	21914	0	3.58E-05	0.00049313	1	8	2
NonTargetingControlGuideForHuman_0045	2	0.99996	0.99971	0.999764	21913	0	3.82E-05	0.00051075	1	9	2
DDO	6	0.26344	0.052691	0.168552	6785	1	6.27E-05	0.00074478	1	10	5
SLC36A1	6	0.93076	0.7074	0.76749	20193	0	6.34E-05	0.00076511	1	11	3
NonTargetingControlGuideForHuman_0005	2	0.99993	0.99953	0.999669	21912	0	7.03E-05	0.00087399	1	12	2
ADORA1	6	0.012939	0.00030338	0.008504	780	2	7.29E-05	0.00090607	1	13	3
MEMO1	6	0.7397	0.34785	0.477227	15957	0	8.26E-05	0.0010624	1	14	2
NonTargetingControlGuideForHuman_0731	2	0.99991	0.99934	0.999519	21911	0	9.17E-05	0.0012232	1	15	2
GLDC	6	0.54405	0.1885	0.348123	11662	1	0.00011866	0.0015476	1	16	4
WFDC9	6	0.93738	0.73245	0.788294	20355	0	0.00012154	0.0016072	1	17	2
NonTargetingControlGuideForHuman_0476	2	0.99987	0.99905	0.999283	21910	0	0.00013305	0.001834	1	18	2
NonTargetingControlGuideForHuman_0075	2	0.99986	0.99898	0.999249	21909	0	0.0001414	0.0019755	1	19	2
ZNF565	6	0.93562	0.72376	0.780403	20319	0	0.00014291	0.0020021	1	20	2
C7orf49	6	0.45745	0.13385	0.294779	9951	1	0.00014365	0.0020252	1	21	2
THAP5	6	0.99985	0.99887	0.999193	21908	0	0.00015071	0.0021503	1	22	6
EPS15L1	6	0.16916	0.025647	0.101609	5454	1	0.00015274	0.0021625	1	23	3
NonTargetingControlGuideForHuman_0341	2	0.99984	0.99884	0.999193	21907	0	0.00015887	0.0022529	1	24	2
KIAA2018	6	0.20367	0.027633	0.101609	5952	1	0.0001589	0.0022529	1	25	3
NonTargetingControlGuideForHuman_0058	2	0.99984	0.99882	0.999193	21906	0	0.00016277	0.0023199	1	26	2
PORCN	6	0.2978	0.063158	0.186469	7296	1	0.0001669	0.0023807	1	27	2
CMA1	6	0.73834	0.34774	0.477227	15930	0	0.00017015	0.00243	1	28	2
NonTargetingControlGuideForHuman_0870	2	0.99982	0.99866	0.999118	21905	0	0.00018104	0.0026527	1	29	2

MAGeCK analysis: gene ranking
Top 30 hits Gemcitabine d14

id	num	neg|score	neg|p-value	neg|fdr	neg|rank	neg|goodsgrna	pos|score	pos|p-value	pos|fdr	pos|rank	pos|goodsgrna
PPP1R32	6	0.034286	0.0026653	0.042143	1269	1	3.39E-06	2.26E-07	0.00015	1	3
hsa-mir-4711	4	0.92423	0.87125	0.945311	20198	0	6.34E-06	2.26E-07	0.00015	2	3
NCOA3	6	0.98785	0.98645	0.999362	21631	0	8.47E-06	2.26E-07	0.00015	3	4
CENPE	6	0.91379	0.85747	0.941615	19944	0	1.52E-05	2.26E-07	0.00015	4	4
UGGT2	6	0.98817	0.98678	0.999369	21638	0	1.61E-05	2.26E-07	0.00015	5	3
ARL1	6	0.44975	0.22345	0.50741	9644	0	2.43E-05	2.26E-07	0.00015	6	1
hsa-mir-548h-5	3	0.99888	0.99966	0.999991	21887	0	3.94E-05	2.26E-07	0.00015	7	2
UBXN2A	6	0.87911	0.79828	0.910972	19200	0	5.76E-05	2.26E-07	0.00015	8	2
NADK2	6	0.092507	0.01462	0.139671	2260	1	7.11E-05	2.26E-07	0.00015	9	2
CLEC19A	6	0.41226	0.18752	0.4618	8836	0	7.29E-05	2.26E-07	0.00015	10	1
PKP4	6	0.9855	0.98288	0.99886	21557	0	7.36E-05	2.26E-07	0.00015	11	4
BGLAP	6	0.27199	0.084905	0.311325	5808	0	9.25E-05	2.26E-07	0.00015	12	3
SHF	6	0.68658	0.50439	0.74486	14810	0	0.00011002	2.26E-07	0.00015	13	4
PDDC1	6	0.9164	0.86066	0.941615	20011	0	0.00012154	2.26E-07	0.00015	14	1
LPPR1	6	0.7502	0.59838	0.806785	16252	0	0.00012822	2.26E-07	0.00015	15	4
LRRC16B	6	0.83209	0.72435	0.874568	18145	0	0.00012888	2.26E-07	0.00015	16	3
MILR1	6	0.16825	0.03779	0.218891	3695	1	0.00013419	2.26E-07	0.00015	17	3
PTOV1	6	0.18487	0.044805	0.225685	4019	1	0.00017015	2.26E-07	0.00015	18	1
TUBA1B	6	0.25114	0.068054	0.274839	5375	1	0.0001829	2.26E-07	0.00015	19	3
hsa-mir-3143	4	0.99962	0.99992	0.999991	21907	0	0.00019384	2.26E-07	0.00015	20	3
RBBP8	6	0.1964	0.045191	0.225685	4260	1	0.00020858	2.26E-07	0.00015	21	2
C1orf141	6	0.23063	0.053314	0.232297	4945	1	0.00021876	2.26E-07	0.00015	22	1
NOLC1	6	0.36999	0.1587	0.437027	7939	0	0.00026737	2.26E-07	0.00015	23	1
FAM13A	6	0.99213	0.99272	0.999991	21737	0	0.00028476	2.26E-07	0.00015	24	3
BVES	6	0.81611	0.70444	0.866016	17787	0	0.00031598	2.26E-07	0.00015	25	1
PCDH18	6	0.87186	0.78798	0.906469	19036	0	0.00034594	2.26E-07	0.00015	26	3
TMEM243	6	0.45067	0.22573	0.50904	9665	0	0.00036458	2.26E-07	0.00015	27	1
TNFRSF25	6	0.1258	0.028319	0.20003	2880	1	0.00037039	2.26E-07	0.00015	28	3
hsa-mir-3195	4	0.33703	0.12538	0.378989	7247	0	0.00037246	2.26E-07	0.00015	29	2

MAGeCK analysis: gene ranking
Top 30 hits Gemcitabine d21

id	num	neg|score	neg|p-value	neg|fdr	neg|rank	neg|goodsgrna	pos|score	pos|p-value	pos|fdr	pos|rank	pos|goodsgrna
TSC2	6	0.93806	0.83211	0.895286	20360	0	6.01E-06	2.26E-07	0.00495	1	4
CRYGC	6	0.93828	0.83225	0.895286	20372	0	1.97E-05	1.13E-06	0.008663	2	4
ATRNL1	6	0.043646	0.00055186	0.008446	1430	1	2.43E-05	1.58E-06	0.008663	3	1
SH3BP4	6	0.53277	0.21048	0.409668	11242	0	2.94E-05	1.58E-06	0.008663	4	4
hsa-mir-6727	4	0.6142	0.31463	0.529915	12973	0	4.86E-05	2.48E-06	0.010891	5	1
MAP4K5	6	0.98361	0.95938	0.978212	21493	0	7.11E-05	3.39E-06	0.011386	6	2
hsa-mir-1254-2	4	0.10667	0.0070364	0.060403	2481	1	0.00010817	4.29E-06	0.011386	7	2
NonTargetingControlGuideForHuman_0555	2	0.99988	0.99996	1	21911	0	0.00011641	4.74E-06	0.011386	8	2
hsa-mir-1265	4	0.99997	0.99999	1	21914	0	0.00011646	4.74E-06	0.011386	9	3
CDH20	6	0.44623	0.14132	0.331057	9355	0	0.00011803	5.20E-06	0.011386	10	4
LHX6	6	0.90563	0.75886	0.849053	19585	0	0.00012154	6.10E-06	0.012151	11	1
OGN	6	0.24413	0.034297	0.144563	5108	1	0.00017015	1.20E-05	0.014407	12	1
MOB1A	6	0.10432	0.0069876	0.060403	2442	1	0.00017699	1.20E-05	0.014407	13	3
hsa-mir-3192	4	0.76671	0.50427	0.675986	16342	0	0.00017825	1.20E-05	0.014407	14	1
HFM1	6	0.96551	0.91266	0.95012	21051	0	0.00017962	1.20E-05	0.014407	15	3
OR5R1	6	0.41494	0.11795	0.297278	8693	0	0.00018167	1.20E-05	0.014407	16	2
FUT1	6	0.43605	0.13047	0.31136	9130	0	0.00020359	1.38E-05	0.014407	17	3
IMPA1	6	0.23495	0.033543	0.144563	4919	1	0.00021765	1.47E-05	0.014407	18	2
NOTUM	6	0.14073	0.015556	0.101451	3094	1	0.00021876	1.47E-05	0.014407	19	1
GPX3	6	0.96943	0.92281	0.956463	21144	0	0.00026082	1.97E-05	0.014407	20	2
TRIM21	6	0.84442	0.62597	0.75589	18146	0	0.00026283	1.97E-05	0.014407	21	3
NUTM1	6	0.14411	0.015614	0.101451	3156	1	0.00027401	2.01E-05	0.014407	22	2
SOCS2	6	0.14287	0.015562	0.101451	3136	1	0.00029533	2.10E-05	0.014407	23	4
HMGCS2	6	0.80267	0.55789	0.711814	17170	0	0.00030053	2.46E-05	0.014407	24	4
LRCH2	6	0.46042	0.15653	0.352398	9663	0	0.00030157	2.46E-05	0.014407	25	3
NonTargetingControlGuideForHuman_0150	2	0.99969	0.99984	1	21909	0	0.00030506	2.51E-05	0.014407	26	2
hsa-mir-145	4	0.94144	0.84493	0.905851	20441	0	0.00031241	2.51E-05	0.014407	27	3
BCOR	6	0.28064	0.046821	0.173201	5871	0	0.00031598	2.51E-05	0.014407	28	1
hsa-mir-4529	4	0.79585	0.54835	0.705037	17010	0	0.000321	2.55E-05	0.014407	29	3

Table S1: MAGeCK analysis gene ranking for NUC-1031 and gemcitabine at d14 and d21.

Primers PCR#1	Primer Sequences	Purification	Synthesis scale (µM)
v2Adaptor_F (Forward)	5'- AATGGACTATCATATGCTTACCGTAACTTGAAAGTATTTCG -3'	HPLC (High performance Liquid chromatography)	0.05
v2Adaptor_R (Reverse)	5'- TCTACTATTCTTTCCCCTGCACTGTTGTGGGCGATGTGCGCTCTG -3'	HPLC	0.05

Primers PCR2	Primer Sequences	Purification	Synthesis scale (µM)
Illumina F1	5'-AATGATACGGCGACCACCGAGATCTACACTCTTTCCCTACACGACG CTCTTCCGATCTTAAGTAGAGTCTTGTGGAAAGGACGAAACACCG-3'	HPLC	0.05
Illumina F2	5'-AATGATACGGCGACCACCGAGATCTACACTCTTTCCCTACACGACG CTCTTCCGATCTATACACGATCTCTTGTGGAAAGGACGAAACACCG-3'	HPLC	0.05
Illumina F3	5'-AATGATACGGCGACCACCGAGATCTACACTCTTTCCCTACACGACG CTCTTCCGATCTGATCGCGCGGTTCTTGTGGAAAGGACGAAACACCG-3	HPLC	0.05
Illumina F4	5'-AATGATACGGCGACCACCGAGATCTACACTCTTTCCCTACACGACG CTCTTCCGATCTCGATCATGATCGTCTTGTGGAAAGGACGAAACACCG-3'	HPLC	0.05
Illumina R1	5'-CAAGCAGAAGACGGCATACGAGATAAGTAGAGGTGACTGGAGTTCA GACGTGTGCTCTTCCGATCTTTCTACTATTCTTTCCCCTGCACTGT-3'	HPLC	0.05
Illumina R2	5'-CAAGCAGAAGACGGCATACGAGATACACGATCGTGACTGGAGTTCA GACGTGTGCTCTTCCGATCTATTCTACTATTCTTTCCCCTGCACTGT-3’	HPLC	0.05
Illumina R3	5'-CAAGCAGAAGACGGCATACGAGATCGCGCGGTGTGACTGGAGTTCA GACGTGTGCTCTTCCGATCTGATTCTACTATTCTTTCCCCTGCACTGT- 3	HPLC	0.05
Illumina R4	5'-CAAGCAGAAGACGGCATACGAGATCATGATCGGTGACTGGAGTTCA GACGTGTGCTCTTCCGATCTCGATTCTACTATTCTTTCCCCTGCACTGT-3'	HPLC	0.05

	Sample	Forward primer	Reverse primer
1	Replicate 1: Baseline	Illumina F1	Illumina R1
2	Replicate 1: Day 14 Control	Illumina F1	Illumina R2
3	Replicate 1: Day 14 Gemcitabine	Illumina F1	Illumina R3
4	Replicate 1: Day 14 Acelarin	Illumina F1	Illumina R4
5	Replicate 1: Day 21 Control	Illumina F2	Illumina R1
6	Replicate 1: Day 21 Gemcitabine	Illumina F2	Illumina R2
7	Replicate 1: Day 21 Acelarin	Illumina F2	Illumina R3
8	Replicate 2: Baseline	Illumina F4	Illumina R4
9	Replicate 2: Day 14 Control	Illumina F3	Illumina R1
10	Replicate 2: Day 14 Gemcitabine	Illumina F3	Illumina R2
11	Replicate 2: Day 14 Acelarin	Illumina F3	Illumina R3
12	Replicate 2: Day 21 Control	Illumina F3	Illumina R4
13	Replicate 2: Day 14 Gemcitabine	Illumina F4	Illumina R1
14	Replicate 2: Day 14 Acelarin	Illumina F4	Illumina R2

PCR#1 Conditions			
Initial denaturation	95˚C	2 min	1 cycle
Denaturation	95˚C	10-20 s	
Annealing	60˚C	20s	24 cycles
Elongation	72˚C	30s	
Final extension	72˚C	3 min	1 cycle
	END		

PCR#2 Conditions			
Initial denaturation	95˚C	2 min	1 cycle
Denaturation	95˚C	15 s	
Annealing	50˚C	20s	18 cycles
Elongation	72˚C	30s	
Final extension	72˚C	3 min	1 cycle
	END		

Table S2: PCR primers and conditions

Characteristics	Number of patients	Per cent %
Gender	85	100
Male	46	54
Female	39	46
Age (years)		
Median	66	-
Range	43 - 84	-
Pathologic stage		
T1	3	3.5
T2	6	7
T3	74	87
T4	2	2.5
N0	15	17.5
N1	68	80
NK	2	2.5
MX	31	36.5
M1	2	2.5
NK	52	61
Tumour differentiation		
Moderate	36	42.5
moderate-poor	11	13
Poor	28	33
well	10	11.5
Treatment		
No chemotherapy	19	22.5
Gemcitabine	24	28
Chemotherapy	14	16.5
Not known	28	33

Table S3: TMA details of pancreatic cancer cohort

Category	Subcategory	Total collective (%)
Sex	Female	27 (72.97)
	Male	10 (27.03)
Age		31 – 83
		median = 56
Tumour type	Ovary	10 (27.03)
	Colon	6 (16.22)
	Oesophageal	3 (8.11)
	Breast	3 (8.11)
	Lung	2 (5.4)
	Endometrium	2 (5.4)
	Pancreas	2 (5.4)
	Biliary	2 (5.4)
	Mesothelioma	2 (5.4)
	Uterine carcinoma	1 (2.7)
	Osteosarcoma	1 (2.7)
	Cervix	1 (2.7)
	Adrenal	1 (2.7)
	Anal	1 (2.7)
Prior gemcitabine	Yes	14 (37.84)
	No	23 (62.16)
RECIST	Partial response (unconfirmed)	2 (5.4)
	Stable disease	25 (67.57)
	Progressive disease	10 (27.03)

Table S4: pan-cancer phase I cohort details

Uncropped gel blots:

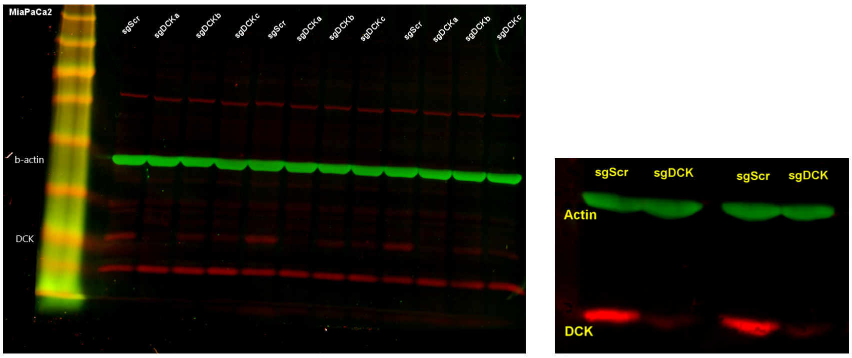

Blots from Fig. 3.

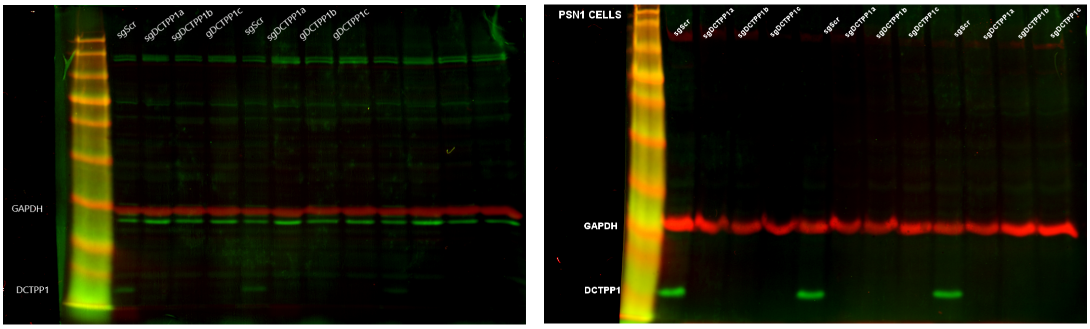

Blots from Fig. 4.

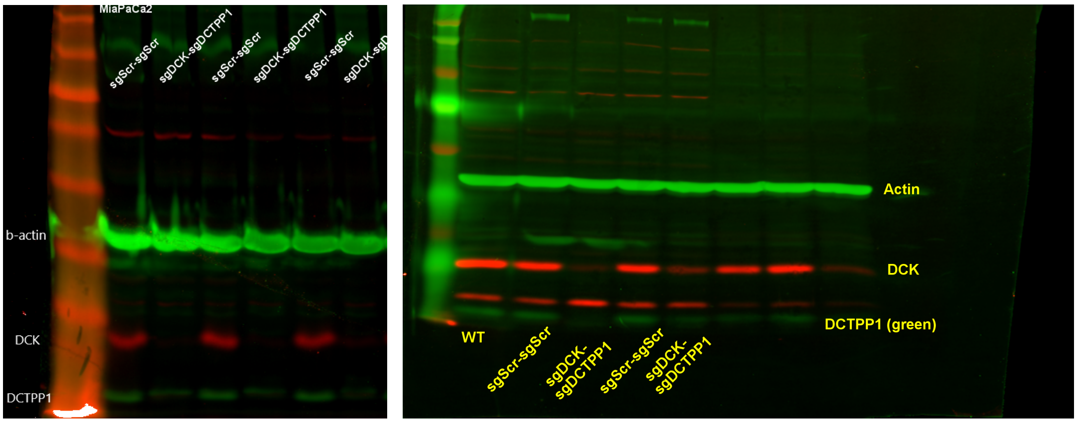

Blots from Fig. 5.
